# Chip-integrated metasurface-enabled single-photon skyrmion sources

**DOI:** 10.1038/s41467-026-75516-5

**Published:** 2026-07-11

**Authors:** Xujing Liu, Yinhui Kan, Shailesh Kumar, Liudmilla F. Kulikova, Valery A. Davydov, Viatcheslav N. Agafonov, Sergey I. Bozhevolnyi

**Affiliations:** 1 Centre of Nano Optics, University of Southern Denmark, Odense, Denmark; 2 Niels Bohr Institute, University of Copenhagen, Copenhagen, Denmark; 3 L.F. Vereshchagin Institute for High Pressure Physics, Russian Academy of Sciences, Moscow, Russia; 4 GREMAN, CNRS, UMR 7347, INSA CVL, Université de Tours, Tours, France

**Keywords:** Nanophotonics and plasmonics, Quantum optics, Nanophotonics and plasmonics, Metamaterials, Optical properties of diamond

## Abstract

Skyrmions, topologically stable field configurations, have recently emerged in classical optics as structured light for high-density data applications. Achieving controllable on-chip generation of single-photon skyrmions, while being highly desirable for quantum information technologies, remains challenging due to the nanoscale confinement of quantum emitters. Here we demonstrate a metasurface-integrated quantum emitter platform enabling room-temperature on-chip generation of single-photon skyrmions. Near-field coupling between quantum emitter and propitiously designed surface arrays of meta-atoms mediates spin-orbit interaction, transforming nanoscale-localized dipole emission into free-propagating topologically structured photonic modes. By exploiting this approach for structuring quantum emission from different color centers in nanodiamonds, we realize diverse skyrmionic states, including high-order anti-skyrmions and skyrmionium, and thereby demonstrate its universality across quantum emitters. Our work establishes a unified framework for on-chip structured quantum light sources, offering versatile control of high-dimensional topological states, such as skyrmions, and advancing scalable quantum photonic technologies.

## Abstract (web summary)

On-chip generation of single-photon skyrmions is challenging due to the nanoscale confinement of quantum emitters. Here the authors demonstrate a metasurface-integrated quantum emitter platform enabling room-temperature on-chip generation of single-photon skyrmions without requiring magnetic fields.

## Introduction

Skyrmions, first introduced in the 1960s as topological solitons in nonlinear field theory^1,2^, have since been predicted and observed across diverse physical systems, including magnetic materials^3,4^, condensate systems^5^, and liquid crystals^6^. By virtue of their swirling textures and quantized topological charges, skyrmions enable robust information encoding in data storage, transfer, and processing^7–10^. In recent years, skyrmions have been extended into the optical domain, ranging from localized electromagnetic fields^11–13^ to propagating vector beams with spatially varying polarization patterns^14–17^. The free-space skyrmionic beams exhibit unique features, including space-time nonseparability and topological resilience in disordered media or free-space propagation^18–20^. More recently, integrated optical platforms have also been explored for generating skyrmionic light, including micro-rings^21^, q-plates^22^, and metasurfaces^23,24^. Despite these advances in classical optics^25–27^, the extension of such topological field configurations to the quantum regime remains at an early stage.

Realizing skyrmionic textures at the single-photon level could unlock previously inaccessible degrees of freedom associated with spatially structured polarization, thereby enriching the landscape of high-dimensional quantum information processing and enhancing robustness against decoherence and environmental noise^28–30^. However, realizing quantum skyrmions in integrated photonic platforms without relying on complex optical components or stringent operating conditions remains highly challenging, particularly for nanoscale solid-state quantum emitters (QEs)^31,32^. The primary obstacle lies in achieving efficient light-matter interactions within confined geometries while simultaneously generating controllable topological features at the subwavelength scale. Therefore, overcoming these limitations necessitates a scalable approach that integrates quantum light emission with topological field control in a compact, chip-based platform.

In this work, we demonstrate the chip-scale generation of single-photon skyrmions using a metasurface-integrated quantum emitter (metaQE) platform. In our approach, light-matter interactions between the QE and the metasurface are mediated by nonradiative, QE-excited surface plasmon polaritons (SPPs), enabling a flexible control of spin-orbital coupling of single-photon emission. By engineering arrays of subwavelength meta-atoms, we realize diverse skyrmionic single-photon states from QEs without relying on bulky optical systems, external magnetic fields, or cryogenic operation, thereby establishing a versatile on-chip platform for generating and exploring topologically structured quantum light for advanced quantum technologies.

## Results

### MetaQE for spin-orbit coupled quantum emission

**Fig. 1:**
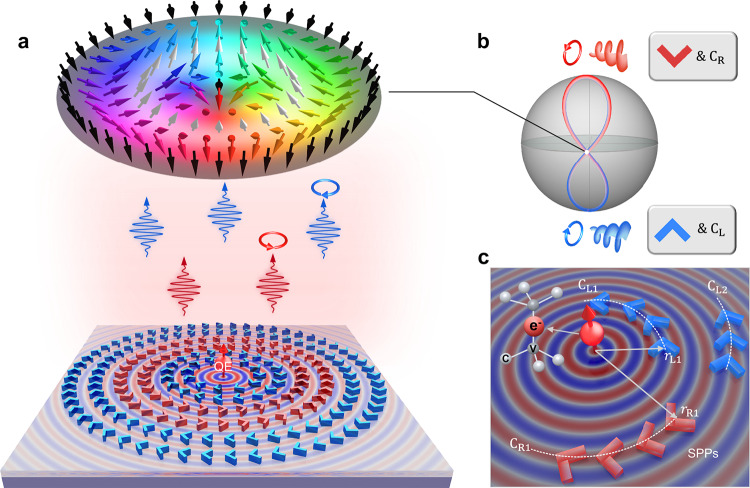
The schematic design of single-photon skyrmion emission from metaQE. **a** Illustration of metaQE emitting single photons with skyrmionium textures. Limited spatial dimensions of a nanoscale QE are extended by QE-excited surface plasmon polaritons (SPPs), which are outcoupled by an on-chip metasurface to free-space radiation with skyrmionic topology. The skyrmion texture is holistically visualized by the arrows showing Stokes vectors. **b** With a strategically designed metasurface, the photon emission features orthogonal spatial and polarization modes, forming a pair of spin-orbit vectorial states represented on a higher-order Poincaré sphere (HOPS). **c** Design principle illustrating that the meta-atom chirality controls spin angular momentum (SAM), and the azimuthal arrangement ${{{{\rm{C}}}}}_{{{{\rm{R}}}}({{{\rm{L}}}})n}$ determines the spatial mode and orbital angular momentum (OAM). The QE features a radiative electrical dipole that is predominantly oriented normal to the surface, thereby efficiently exciting radially diverging, in-plane propagating SPPs. The inset shows a schematic atomic structure of the color center. The red and blue meta-atoms with opposite chiralities outcouple the SPPs into right- and left-circularly polarized (RCP and LCP) emission, respectively. ${r}_{{{{\rm{R}}}}1({{{\rm{L}}}}1)}$ denotes the initial radii of the corresponding trajectories.

At-source manipulation of QE emission is inherently challenging due to QE nanoscale dimensions, thus leaving very limited space for shaping light-matter interactions^33^. To overcome this limitation, the developed metasurface-based approach enables nonradiative QE excitation of in-plane spreading surface electromagnetic waves, i.e., QE-excited SPPs^34^. A QE is located atop the 20 nm silica (SiO_2_) and 150 nm silver (Ag) films and surrounded by an ultrathin, carefully designed and fabricated dielectric metasurface. Upon excitation, the QE generates an electromagnetic dipole primarily polarized normal to the surface, which couples efficiently to nonradiative SPPs propagating radially along the dielectric-metal interface^35^. Here, QE-excited SPPs act as an intermediate channel between the nanoscale emitter and the metasurface, providing exceptional freedom for at-source tailoring spatially distributed phase and polarization of quantum emission. This operating principle is fundamentally different from previous metasurface studies based on externally incident free-space light^36,37^. With properly designed individual subwavelength-sized metasurface elements, meta-atoms, the QE-excited SPPs are outcoupled to free-space radiation with engineered spin-orbit textures, thereby generating the desired skyrmionic single-photon emission (Fig. 1a).

The skyrmionic beam features a spatially varying polarization texture that wraps the Poincaré sphere. Here, the composite metasurface is composed of two surface domains containing meta-atoms of opposite chirality, with the chirality of meta-atoms controlling the spin angular momentum (SAM) components of emitted photons and their spatial arrangement controlling the corresponding orbital angular momentum (OAM) and spatial distribution. In this way, the metasurface constructs two spatially distributed orthogonal basis modes, represented as the poles of a higher-order Poincaré sphere (HOPS), whose superposition gives rise to the skyrmionic topology (Fig. 1b). This goes beyond the generation of single-photon emission with selected polarizations or OAMs^30,34^, requiring instead a conceptually different design strategy that establishes well-defined spatial amplitude, phase, polarization, and radial-mode relations between the two basis modes that enable the local Stokes vector to continuously evolve in the skyrmionic beam cross section, uniformly covering the Poincaré sphere. To tailor the SAM, we employ the L-shaped meta-atoms oriented at $\pm 45^\circ$ with respect to the SPP propagation direction (colored by red and blue). The structural anisotropy (Fig. 1c) enables selective outcoupling of the QE-excited SPPs into free-space photon emission with right- and left-handed circular polarizations (RCP and LCP). The detailed design of the meta-atom is presented in Supplementary Note 1. The OAM is characterized by the vortex topological charge ${\ell}$ that is related to the 2π${\ell}$ phase increment around the vortex core. To accurately manipulate the phase wavefront, the meta-atoms are arranged in various trajectories ${{{{\rm{C}}}}}_{{{{\rm{R}}}}({{{\rm{L}}}})}$ (e.g., concentric or spiral) for accommodating the spatially varying phase of scattering fields, thereby generating photon emissions with Laguerre-Gaussian (LG) modes, $|{{{{\rm{LG}}}}}_{p}^{{\ell}}{{\rangle }}$. The topological charge ${\ell}$ is determined by the number of spiral arms m, following the spin-orbit relation ${{\ell}}_{{{{\rm{R}}}}({{{\rm{L}}}})}=m\pm 1$^38,39^. Meanwhile, the radial index p is dictated by the number of trajectories ${{{{\rm{C}}}}}_{{{{\rm{R}}}}({{{\rm{L}}}})n}\,(n={{\mathrm{1,2,3}}}...)$, representing how many annular rings occupied alternatively with the RCP(LCP)-type meta-atoms are used. Increasing n introduces additional radial nodes in the field amplitude, corresponding to higher-order LG modes. In this way, both the azimuthal and radial mode orders of the emitted photons are controlled by the metasurface configuration, forming different LG modes with orthogonal polarizations. Consequently, the metaQE converts the nm-localized QE excitation into a free-space single-photon emission with spatially varying polarization and phase distributions, thereby enabling on-chip generation of single-photon skyrmions. We note that in a recent study^31^ reporting an important realization of single-photon skyrmions based on a quantum dot coupled to a Gaussian microcavity, mode selectivity was achieved through Zeeman splitting under low temperature and an external magnetic field. In contrast, our chip-integrated metasurface-QE platform is based on a different physical mechanism and operating regime, enabling room-temperature operation without external magnetic fields and providing flexible access to a broader range of skyrmionic states through the dedicated metasurface design.

### Experimental realization of anti-type skyrmion

**Fig. 2:**
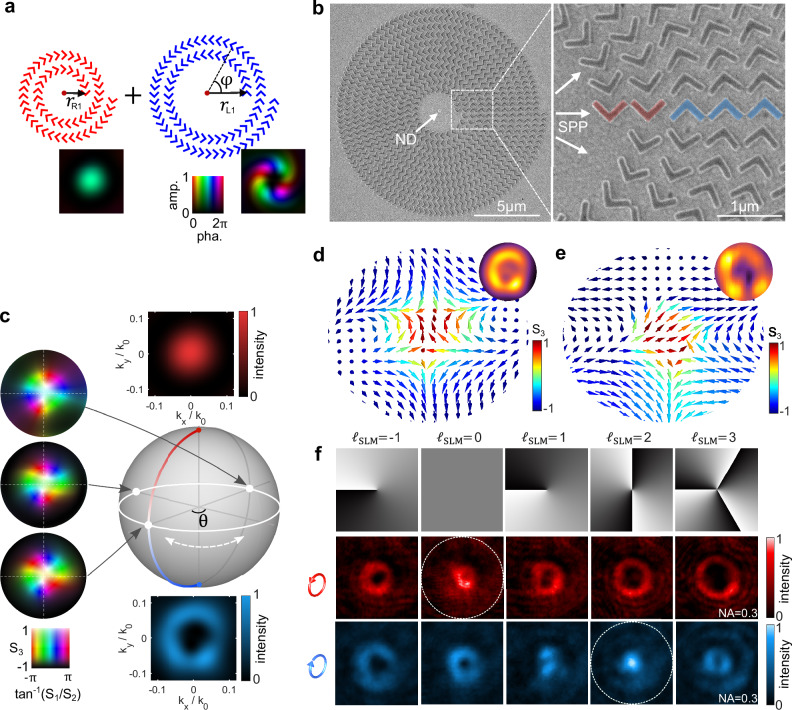
Anti-type skyrmion photon emission from nitrogen-vacancy (NV) centers in nanodiamond. **a** Metasurface design for funneling QE emission to two eigenstates of Laguerre-Gaussian (LG) mode. Meta-atoms of opposite chirality follow spiral trajectories with different initial radii, thereby generating RCP emission with ${{\ell}}_{{{{\rm{R}}}}}=0$ and LCP emission with ${{\ell}}_{{{{\rm{L}}}}}=-2$. Inset: the far-field spatial phase distribution, the hue represents the phase, and the lightness denotes the intensity. **b** SEM image of NV centers integrated with a metasurface, where the magnified SEM image alongside highlights the varying chirality of the meta-atom for controlling SAM. **c** Evolution of skyrmionic emission with different helicities by tuning the initial radius. The poles of HOPS correspond to two eigenstates of the LG mode; the angular coordinate θ denotes the relative phase between the modes controlled by the start radius. **d** Simulated and **e** measured skyrmion map from the emitted photons, in which the vector arrows denote the spin azimuthal of Stokes vectors and the color indicates circular polarized components ${S}_{3}$. **f** Verification of the topological charge of the RCP and LCP components of the photon emission. Polarization-resolved far-field emission patterns projected to SLM holograms with ${{\ell}}_{{{{\rm{SLM}}}}}=-{{\mathrm{1,0,1,2,3}}}$.

Figure 2a shows the metaQE design to construct skyrmionic photon emission with orthogonal polarizations and distinct LG modes. The two types of meta-atoms are arranged along Archimedean spiral trajectories: ${r}_{{{{\rm{R}}}}\left({{{\rm{L}}}}\right)}(\varphi )={r}_{{{{\rm{R}}}}\left({{{\rm{L}}}}\right)1}+m{\lambda }_{{{{\rm{SPP}}}}}\frac{\varphi }{2{{{\rm{\pi }}}}}$, where φ is the azimuthal angle of each meta-atom, m is the number of spiral arms (here m=1), ${r}_{{{{\rm{R}}}}1}$ and ${r}_{{{{\rm{L}}}}1}$ define the initial radii of the respective trajectories, and ${\lambda }_{{{{\rm{SPP}}}}}$ is the propagation wavelength of SPPs (estimated by the filling ratio of metasurface and the QE emission wavelength^34^). Hence, combining the two metasurfaces can simultaneously modulate QE emission to produce ${{{{\rm{LG}}}}}_{0}^{0}({{\ell}}_{{{{\rm{R}}}}}=0,\,p=0)$, mode with RCP and ${{{{\rm{LG}}}}}_{0}^{-2}({{\ell}}_{{{{\rm{L}}}}}=-2\,p=0)$, mode with LCP, given by^27^:

1 $$|{{{\mathbf{\Psi }}}}\left(r\right){{\rangle }}={{{{\rm{LG}}}}}_{0}^{0}\left(r\right)|{{{\rm{R}}}}{{\rangle }}+\exp \left(i\theta \right){{{{\rm{LG}}}}}_{0}^{-2}\left(r\right)|{{{\rm{L}}}}{{\rangle }}$$

in which$\,\theta$ denotes the phase difference between the two modes, $|{{{\rm{R}}}}{{\rangle }}$ and $|{{{\rm{L}}}}{{\rangle }}$ represent RCP and LCP states. The spatial and azimuthal profiles of OAM modes (inset of Fig. 2a) reveal that RCP component features a Gaussian beam with ${{\ell}}_{{{{\rm{R}}}}}=0$, whereas LCP possesses azimuthal phase of ${{\ell}}_{{{{\rm{L}}}}}=-2$, forming a doughnut-shape intensity distribution. In the experiment, the metasurface (made of hydrogen silsesquioxane, HSQ) was precisely integrated with a nanodiamond (ND) containing nitrogen-vacancy (NV) centers. The fabrication was facilitated by high-precision alignment markers and electron-beam lithography (EBL) techniques (see Supplementary Note 3 for details), enabling a positioning accuracy within 50 nm. The scanning electron microscopy (SEM) image of the device (Fig. 2b) shows that the meta-atoms have a width of 100 nm, a length of 400 nm, and are arranged with a fixed period of 580 nm (${\lambda }_{{{{\rm{SPP}}}}}$) along the radial direction. The precision in relative lateral positioning of the ND with respect to the metasurface is estimated to be ~20 nm. The false color rendering highlights the opposite chirality of the meta-atoms, which form spirals with winding numbers of 2 and 7, respectively. The outer region is set with more winding numbers to compensate for the attenuation of SPPs during their radial propagation. The structure ultimately forms nonseparable spin-orbital states at the pole of HOPS, thereby forming a synthesized optical skyrmion situated at the HOPS equator (Fig. 2c). The skyrmion topology is revealed by the polarization states characterized by the Stokes parameters (${S}_{0},\,{S}_{1},\,{S}_{2},\,{S}_{3}$), in which the hue color visualizes the azimuth of transverse $({S}_{1},\,{S}_{2})$ component and lightness (from black to white) indicates the circular polarization component ${S}_{3}$ variation (from RCP to LCP). The topological property is characterized by the skyrmion number defined as:

2 $${N}_{{{{\rm{sk}}}}}=\frac{1}{4\pi }\iint {{{\bf{S}}}}\cdot\left(\frac{\partial {{{\bf{S}}}}}{\partial x}\times \frac{\partial {{{\bf{S}}}}}{\partial y}\right){{{\rm{d}}}}x{{{\rm{d}}}}y$$

with the Stokes vector ${{{\bf{S}}}}=({S}_{1},{S}_{2},{S}_{3})/{S}_{0}$, a nontrivial number ${N}_{{{{\rm{sk}}}}}$ indicates the number of times the polarization vectors cover the full Poincare sphere. Here, the integration domain for evaluating the skyrmion number is defined as the circular region where the total far-field intensity exceeds $1/{e}^{2}$ of its maximum value, a criterion that is widely used for evaluating the Gaussian beam diameter. The obtained polarization field indicates the higher-order anti-type skyrmion distributions with the simulated skyrmion number of −1.98. Moreover, another prominent feature of this design is that the skyrmion distribution can be independently controlled. The intensity and phase determine the latitude and longitude of HOPS, respectively. By varying the initial radii between the two spirals ($\Delta r={r}_{1}-{r}_{2}$), the relative phase θ between the two modes can be continuously tuned from 0 to 2π along the equator of HOPS, following $\theta=2\pi \Delta r/{\lambda }_{{{{\rm{SPP}}}}}$. This relative phase directly maps onto the initial phase γ of the azimuthal vector distribution, enabling full coverage of all possible orientations of the helical field. The three representative cases shown in Fig. 2c illustrate this behavior: choosing Δr of 0, $\frac{\pi }{4}{\lambda }_{{{{\rm{SPP}}}}}$, and $\frac{\pi }{2}{\lambda }_{{{{\rm{SPP}}}}}$, produces relative phase shifts of 0, $\frac{\pi }{2}$, π, which results in rotation of the anti-skyrmion distribution of $\gamma=\pi,\,\frac{\pi }{2}$ and 0. For skyrmionic configurations with positive ${N}_{{{{\rm{sk}}}}}$ $({{{\rm{e}}}}.{{{\rm{g}}}}.\,{{\Delta }}{\ell}=1)$, tuning Δr also allows a full transition between Néel-type $(\gamma=0,\pi )$ and Bloch-type $(\gamma=\pm \pi /2)$ skyrmions, thereby providing complete control over the skyrmion helicity (Supplementary Note 2).

To efficiently excite the vertical dipole transition of the NV centers, we employed a 532 nm continuous-wave radially polarized laser, providing a dominant *z*-oriented electric-field component. The polarization-resolved far-field emission was then analyzed by measuring the Stokes parameters using a quarter-wave plate and a linear polarizer (experimental setup in Supplementary Note 4). The skyrmion topology is illustrated by vectorial arrows, where the directions are derived from the Stokes parameters via arctan$({S}_{1}/{S}_{2})$, and the color varies with ${S}_{3}$. Both simulated (Fig. 2d) and measured (Fig. 2e) polarization states show a progressive change of the vector orientation from the up state at the center to the down state at the edge, covering all intermediate polarization states from RCP to LCP. The measured skyrmion number is −1.86, evaluated over an integration region based on the far-field intensity distribution shown in the inset, within a polar angle of 9°. The deviation from the ideal skyrmion number (−2) mainly originates from the non-uniform far-field intensity distribution measured in the experiment, which affects the robustness of the retrieved Stokes parameters in weak-intensity regions. This non-uniformity may arise from the ND position shift, as well as fabrication imperfections. Based on the analysis of the influence of ND positioning accuracy (Supplementary Fig. 8), the skyrmion number decreases monotonically with increasing displacement, while remaining about −1.9 for lateral offsets of 30 nm. Nevertheless, the main skyrmionic textures are preserved within this moderate displacement range, indicating robust tolerance to positioning imperfections. The result reveals the intrinsic intertwining between the spatial pattern and polarization, which is preserved even after the emitted photons pass through multiple optical components, including dichroic mirrors, reflection mirrors, bandpass filters, and imaging lenses. This preservation of the anti-skyrmion distribution, despite multiple reflections and optical transformations, demonstrates the topological protection of the emitted photons and their resilience to optical perturbations. To further analyze the distinct OAM modes, the RCP and LCP components were separately projected onto a spatial light modulator (SLM) encoded with ${{{{\rm{LG}}}}}_{0}^{{{\ell}}_{{{{\rm{SLM}}}}}}$ phase holograms with ${{\ell}}_{{{{\rm{SLM}}}}}=-1,\,0,\,1,\,2,\,3$ (Fig. 2f). Without any additional phase modulation (${{\ell}}_{{{{\rm{SLM}}}}}=0$), the emission appears as a central spot in the RCP channel and a doughnut-shaped pattern in the LCP channel, both being consistent with the targeted spatial mode profiles shown in Fig. 2c. When the OAM of the emitted photon is compensated by the conjugate OAM phase encoded on the SLM, the diffracted beam is converted into a Gaussian-like spot. Accordingly, bright Gaussian spots are observed at ${{\ell}}_{{{{\rm{SLM}}}}}=0$ for the RCP component and at ${{\ell}}_{{{{\rm{SLM}}}}}=2$ for the LCP component, confirming the topological charges of ${{\ell}}_{{{{\rm{R}}}}}=0$ and ${{\ell}}_{{{{\rm{L}}}}}=-2$, respectively. Deviations in the measured OAM mode distribution from the simulated one (Supplementary Fig. 9) may arise from the wavelength-dependent response of the SLM to the broadband emission of NV centers, as well as from minor fabrication imperfections of the component. Furthermore, we have also analyzed the corresponding spatially resolved relative phase distribution between the two spin-orbit components, retrieved from the Stokes parameters^40^, and observed a 4π azimuthal phase winding (Supplementary Fig. 10), providing further support for the targeted skyrmion topology.

### Engineering skyrmionium topology

In general, the skyrmion topology collapses when the skyrmion number approaches zero, indicating a deformation or annihilation of the spin texture^17^. The concept of a skyrmionium refers to a bound pair of a skyrmion and an anti-skyrmion with opposite skyrmion numbers. Although its total skyrmion number is zero, the structure retains a nontrivial internal texture that enables stable, localized motion and suppresses the skyrmion Hall effect^41^. Despite its conceptual importance, the realization of such nontrivial topology in quantum light remains largely unexplored and has not yet been experimentally realized. Inspired by magnetic skyrmionium structures^42^, we aim to engineer the photon emission of QEs to exhibit analogous topological textures in their polarization states, thereby generating propagating single photons carrying a skyrmionium texture. Achieving this requires a tailored coupling between SAM and OAM, which can be constructed by the superposition of the eigenstates of ${{{\rm{L}}}}{{{{\rm{G}}}}}_{0}^{1}$ with no radial mode (${{\ell}}_{{{{\rm{R}}}}}=1$, p=0) and ${{{\rm{L}}}}{{{{\rm{G}}}}}_{1}^{-1}$ with radial mode (${{\ell}}_{{{{\rm{L}}}}}=-1$, p=1), given by:

3 $$|{{{\mathbf{\Psi }}}}\left(r\right){{\rangle }}={{{{\rm{LG}}}}}_{0}^{1}\left(r\right)|{{{\rm{R}}}}{{\rangle }}+\exp \left({{{\rm{i}}}}\theta \right)\,{{{{\rm{LG}}}}}_{\,1}^{-1}\left(r\right)|{{{\rm{L}}}}{{\rangle }}$$

**Fig. 3:**
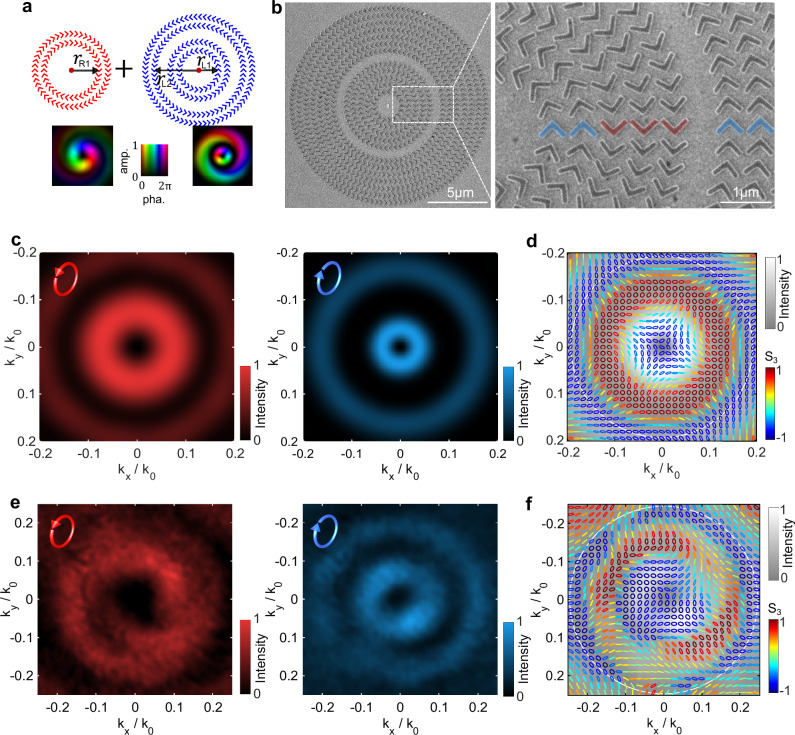
Realization of skyrmionium photon emission from NV centers. **a** Metasurface design for funneling QE emission to two eigenstates of ${{{\rm{L}}}}{{{{\rm{G}}}}}_{0}^{1}|{{{\rm{R}}}}{{\rangle }}$ and ${{{\rm{L}}}}{{{{\rm{G}}}}}_{1}^{-1}|{{{\rm{L}}}}{{\rangle }}$ mode. The meta-atoms are arranged along concentric paths with different starting radii, enabling spatial polarization separation and sequential transitions of OAM. **b** SEM image of metasurface integrated with nanodiamonds hosting NV centers. **c** Simulated and **e** measured far-field intensity profile of RCP and LCP components of the emitted photons. The displayed area corresponds to an NA of 0.2. **d** Simulated and **f** measured polarization states superimposed on the emission intensity. The skyrmion regions are enclosed by white circles, and the ellipses denote the local polarization states, with color representing the Stokes parameter ${S}_{3}$.

The different radial distributions of mode fields reduce the spatial overlap between the RCP and LCP components. For coupling with the local QE, we consider the meta-atoms with opposite chirality arranged along concentric paths $\left(m=0\right)$ with different initial radii ${r}_{{{{\rm{R}}}}1}=2{{{{\rm{\lambda }}}}}_{{{{\rm{SPP}}}}}$, ${r}_{{{{\rm{L}}}}1}=4{{{{\rm{\lambda }}}}}_{{{{\rm{SPP}}}}}$, and ${r}_{{{{\rm{L}}}}2}=6.75{{{{\rm{\lambda }}}}}_{{{{\rm{SPP}}}}}$, as shown in Fig. 3a. The configuration generates three doughnut-shaped emission beams with distinct divergence angles, corresponding to controlled coupling of the QE-excited SPPs into multiple OAM channels. The spatial phase profile shows a clear spatial polarization separation and sequential transition of the dominant OAM states, from ${{\ell}}_{{{{\rm{R}}}}}=1{{\rangle }}$ to ${{\ell}}_{{{{\rm{L}}}}}=-1{{\rangle }}$, and back to ${{\ell}}_{{{{\rm{R}}}}}=1{{\rangle }}$ across the beam cross section, satisfying the conditions required for a skyrmionium polarization texture. Figure 3b shows the SEM image of the fabricated metasurface integrated with a nanodiamond containing NV centers. The false color rendering highlights the rotation of meta-atoms that control the SAM of the emitted photons, and the concentric starting radii tune the accessible range of OAM. The measured far-field intensity of RCP and LCP components reveals three well-separated doughnut-shaped beams with distinct divergence angles, in excellent agreement with numerical simulations (Fig. 3c, e). A weak outer ring seen in the RCP intensity distribution may originate from residual SPPs in the gap between neighboring rings of opposite handedness, and can progressively be suppressed as the gap is reduced (for example, by decreasing ${r}_{{{{\rm{L}}}}2}$; Supplementary Fig. 11) or as the number of RCP rings (Supplementary Fig. 12) is increased, owing to more efficient SPP outcoupling. The spatial evolution of the polarization ellipses further displays a continuous transformation from RCP to LCP and back to RCP as the radial position increases (Fig. 3d, f), confirming the nested skyrmion and anti-skyrmion configuration characteristic of a skyrmionium. The measured net skyrmion number of 0.07 closely matches the simulated value of 0.05, with the slight deviation from zero attributed to non-uniform emission intensity and measurement noise. These results demonstrate a neutral yet topologically structured polarization field with a skyrmionium topology. Moreover, as discussed in Fig. 2c, varying the initial radii of the meta-atoms simply shifts the relative phase and thus rotates the skyrmionium azimuthally (Supplementary Fig. 11).

### Skyrmionium single-photon sources with GeV centers

**Fig. 4:**
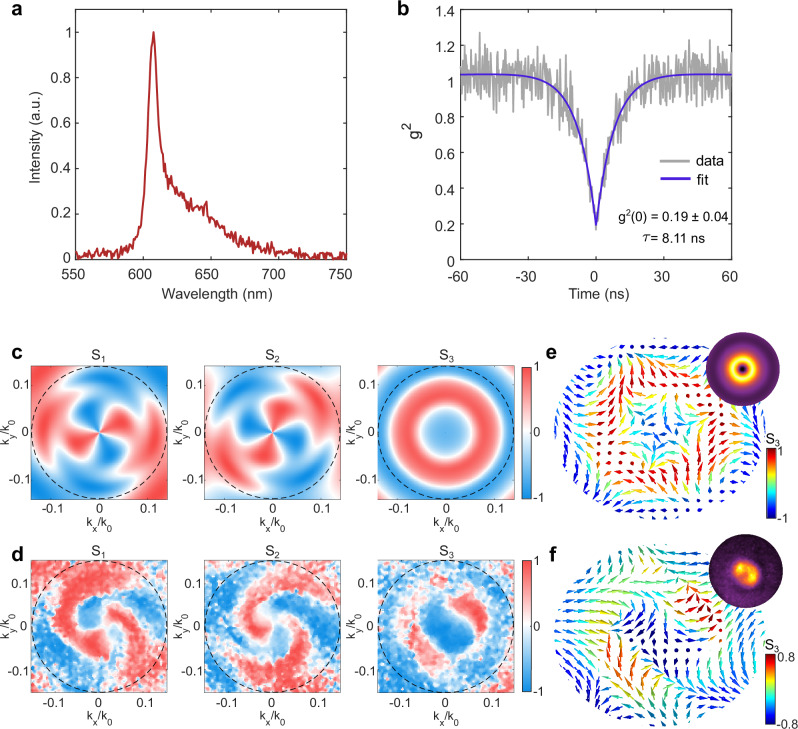
Single-photon skyrmionium emission from the GeV center. **a** Photoluminescence spectrum from the metasurface integrated GeV center. Under a continuous-wave excitation power of 100 μW, the detected photon rate reaches $7\times {10}^{4}$ counts/s. **b** Second-order correlation ${{{{\rm{g}}}}}^{2}(\tau )$ measured at room temperature. The lifetime is 8.11 ns. **c** Simulated and **d** measured Stokes parameters of the single photon emission. **e** Simulated and **f** measured polarized distribution of the emitted single photons, exhibiting a complete transition from RCP to LCP back to RCP. The integration region used to calculate the skyrmion number follows the corresponding far-field intensity profiles, with NA = 0.14 (**e**) and NA = 0.15 (**f**).

To demonstrate the generality of the approach, we applied the metasurface design to single germanium-vacancy (GeV) centers in nanodiamonds, which is particularly appealing for quantum technologies due to their high brightness, narrow linewidth, and exceptional spectral stability^43,44^. In the experiment, we preselected nanodiamonds hosting a single GeV center with a dominant vertically oriented transition dipole^45^. The metasurface period was optimized to 490 nm (${\lambda }_{{SPP}}$) to match the GeV-excited SPP mode. The photoluminescence spectrum of the device features a pronounced emission peak at 602 nm (Fig. 4a). The second-order correlation measurement in a Hanbury Brown-Twiss configuration yields *g*^2^(0) = 0.19 ± 0.04, confirming the single-photon emission at room temperature (Fig. 4b). The measured Stokes parameters show two successive SAM reversals, in excellent agreement with simulation (Fig. 4c, d). The measured polarization state further confirms the skyrmionium-type topology, exhibiting the characteristic pair of oppositely oriented spin-polarization lobes (Fig. 4e, f). The skyrmion number is 0.06 for the single-photon emission. The minor deviations and the slight rotation of the Stokes pattern may arise from fabrication-induced asymmetries of constituent meta-atoms and an imperfect (i.e., deviating from normal to the surface plane) radiative dipole orientation of the color center, which lead to unequal RCP/LCP amplitudes and an intensity discontinuity at the lobe boundary (inset of Fig. 4f). In addition, inherently weak single-photon signals may further be attenuated after passing through multiple optical components during the measurement procedure, making the above effects more pronounced. Nevertheless, the nontrivial skyrmion texture remains clearly visible, indicating that the proposed design can reliably preserve the key skyrmionic features. Importantly, these results show that skyrmionium single-photon emission can be readily realized with different color centers, and the underlying principle is fully compatible with other solid-state emitters, e.g., semiconductor quantum dots and defects in 2D materials. The metaQE design, therefore, provides a general and versatile route toward topologically structured single-photon sources.

## Discussion

In summary, we have demonstrated a nanophotonic quantum light source that brings photonic topology into single-photon emission. By harnessing SPP-mediated light-matter interactions between individual QEs and engineered metasurfaces, we generate flying quantum skyrmions at room temperature, without requiring magnetic fields. The metaQE provides deterministic spin-orbit coupling at the chip scale, facilitating the transfer of the dipole field into different structured topological modes. The experimental realization of anti-skyrmion and skyrmionium photon emissions validates the capability of the platform to generate diverse topologically structured quantum light. Its implementation with distinct color centers further highlights the generality and scalability of the metasurface-based design. Looking ahead, combining our design with advances in the readout fidelity of color centers could enable chip-scale quantum-enhanced sensing and biological imaging^46,47^. More broadly, our work establishes a pathway toward intrinsically robust quantum-information carriers and opens new opportunities for high-dimensional quantum state control.

## Methods

### Device fabrication

Our metaQE devices were fabricated following an established procedure (Supplementary Fig. 5). A 150 nm Ag film was thermally evaporated onto a silicon substrate and coated with a 20 nm SiO₂ spacer by magnetron sputtering. Gold alignment markers were patterned by EBL (JEOL-6490, 30 kV), metal deposition, and lift-off. Nanodiamonds containing color centers (NV or GeV) were dispersed in deionized water at an optimized concentration and spin-coated onto the prepared substrates to obtain spatially isolated NDs. Their positions were identified by dark-field microscopy referenced to the alignment markers. A 150 nm HSQ layer was then spin-coated at 2200 rpm for 45 s and prebaked at 160 °C for 2 min. Subsequently, metasurfaces were written around selected NDs by EBL and developed in 25% TMAH for 4 min, followed by rinsing in deionized water.

### Optical characterization

Optical measurements were carried out using a home-built confocal micro-photoluminescence setup (Supplementary Fig. 6). A radially polarized 532-nm laser beam was tightly focused onto the metaQE using a 100× objective (NA = 0.9, Olympus MPLFLN). The sample was scanned with a three-axis piezoelectric stage to identify the precise position of QE. The emitted fluorescence was collected by the same objective, passed through a dichroic mirror (to filter the incident light), and detected by an avalanche photodiode (APD) for photon count mapping; polarization-resolved emission was measured by inserting a quarter-wave plate and a linear polarizer before detection. Stokes parameters were obtained from sequential measurements with different analyzer settings (Supplementary Note 4); second-order photon correlation measurements were performed using a Hanbury Brown-Twiss interferometer. The fluorescence was divided by a 50:50 non-polarizing beamsplitter and sent to two single-photon APDs. Photon arrival times were recorded using a time-correlated single-photon counting module (PicoHarp 300) operated in start-stop mode. The normalized coincidence histogram yielded the second-order correlation function *g*^2^(*τ*), where *g*^2^(0) < 0.5 confirmed single-photon emission. The uncertainty was estimated by Poisson bootstrap resampling of the coincidence histogram. All measurements were carried out at room temperature.

### Numerical simulations

Three-dimensional finite-difference time-domain simulations were used to model the metaQE. A z-oriented electric dipole source was placed 30 nm above the SiO_2_/Ag substrate and centered within the dielectric nanostructures (height 150 nm, refractive index 1.41). The dipole emission wavelength was set to 670 nm for the NV center and 602 nm for the GeV center to match their emission peaks at room temperature. Perfectly matched layer boundary conditions were applied in all directions. A non-uniform mesh was used with 10 nm resolution around the QE and the metal-dielectric interface. To extract the angular emission pattern, a two-dimensional field monitor was placed 30 nm above the metasurface to record the near-field distribution. The far-field radiation pattern was then obtained by applying a near-to-far-field transformation, yielding the polarization-resolved far-field intensity distributions used for comparison with experimental measurements.

## Supplementary information

Supplementary Information

Transparent Peer Review file

## Data Availability

The data supporting the findings of this study are available both within this article and in the Supplementary Information. The raw data generated in this study are available from the corresponding authors upon request.

## Code availability

The code used for the theoretical design in this study is available in the Code Ocean capsule associated with this article.
