# Supplementary material for: Chip-integrated metasurface-enabled single-photon skyrmion sources
Source: Nat Commun. 2026 Jul 11;17:8567. doi: 10.1038/s41467-026-75516-5 (PMC13483359; doi:10.1038/s41467-026-75516-5)
Supplement: Supplementary file 1 [file 41467_2026_75516_MOESM1_ESM.pdf]

Supplementary Information

Chip-integrated metasurface-enabled single-photon skyrmion sources

Xujing Liu[1], Yinhui Kan[1,2]*, Shailesh Kumar[1], Liudmilla F. Kulikova[3], Valery A. Davydov[3], Viatcheslav N. Agafonov[4], Sergey I. Bozhevolnyi[1]*

[1]Centre of Nano Optics, University of Southern Denmark, Odense, Denmark

[2]Niels Bohr Institute, University of Copenhagen, Copenhagen, Denmark

[3]L.F. Vereshchagin Institute for High Pressure Physics, Russian Academy of Sciences, Moscow, Russia

[4]GREMAN, CNRS, UMR 7347, INSA CVL, Université de Tours, Tours, France

*Corresponding author. email: yinhui.kan@nbi.ku.dk; seib@mci.sdu.dk

Supplementary Note 1 Meta-atom design for quantum emitter polarization control

The quantum emitter (QE) placed near the dielectric-metal surface efficiently launches surface plasmon polaritons (SPPs), which propagate radially on the surface. Our goal is to design the meta-atom that can outcouple these QE-excited SPPs into free-space light with different spin angular momentum at arbitrary locations on the surface. We first consider the scattering of an SPP by a single dielectric nanoscatterer with width w and length L, whose long axis is 45° tilted with respect to the local SPP propagation direction (Supplementary Fig. 1a). The in-plane field for the incident SPP propagating along the x direction is approximated as

$$\mathbf{E}_{\text{SPP}} \propto E_0 \begin{pmatrix} 1 \\ 0 \end{pmatrix} e^{-ik_{\text{SPP}}x}, \tag{1}$$

where E_0 is the complex field amplitude, ω is the angular frequency, $k_{\text{SPP}} = 2\pi/\lambda_{\text{SPP}}$, λ_{SPP} is the propagation wavelength of the SPP wave. The scattered field generates two orthogonal field components, parallel and perpendicular to the long axis, whose amplitudes are governed by the corresponding polarizability tensor of the anisotropic dielectric scatterer. Here, we assume that there is no phase considered and no resonance due to the dielectric nano-scatterers. The structural anisotropy of the meta-atom makes the scattered field along the long axis much larger than that along the short axis, i.e., $E_{\text{scl}} > E_{scs}$, thus one polarization component dominates over the other, providing the basis for linear polarization control.

Circularly polarized light can be generated when two orthogonal linearly polarized field components have comparable amplitudes and a relative phase difference of $\pm\pi/2$. Since the SPP field has the spatial phase form $e^{-ik_{\text{SPP}}x}$, a spatial displacement between two scattering elements naturally produces a controllable phase delay. Based on this principle, we construct the meta-atom from two anisotropic nanobricks with separated distance. A quarter-wavelength separation $\lambda_{\text{SPP}}/4$ is required between the two scattering elements along the SPP propagation direction, which supplies the phase delay $\Delta\varphi = k_{\text{SPP}}\frac{\lambda_{\text{SPP}}}{4} = \frac{\pi}{2}$. This phase delay, together with the anisotropic scattering of each nanobrick, enables the generation of circularly polarized light. If we consider the scattering contribution associated with the long axis of the nanobrick, the scattered field can be written as

$$\mathbf{E}_{scl} \propto C_1 \begin{pmatrix} 1 + e^{-\frac{i\pi}{2}} \\ -1 + e^{-\frac{i\pi}{2}} \end{pmatrix} = C_1 \begin{pmatrix} 1 - i \\ -1 - i \end{pmatrix} = C_1(1 - i)\begin{pmatrix} 1 \\ -i \end{pmatrix} \propto C_1 e^{-\frac{i\pi}{4}} \begin{pmatrix} 1 \\ -i \end{pmatrix} \tag{2}$$

which corresponds to a right-handed circularly polarized (RCP) component propagating in the $+z$ direction. By contrast, the weaker scattering contribution associated with the short axis of the nanobrick is

$$\mathbf{E}_{scs} \propto C_2 \begin{pmatrix} 1 + e^{-\frac{i\pi}{2}} \\ 1 - e^{-\frac{i\pi}{2}} \end{pmatrix} = C_2 \begin{pmatrix} 1 - i \\ 1 + i \end{pmatrix} = C_2(1 - i)\begin{pmatrix} 1 \\ i \end{pmatrix} \propto C_2 e^{-\frac{i\pi}{4}} \begin{pmatrix} 1 \\ i \end{pmatrix} \tag{3}$$

which corresponds to a left-handed circularly polarized (LCP) component propagating in the

$+z$direction. Therefore, the total outcoupled field is predominantly RCP, owing to the strong anisotropy of the dielectric nanobrick, $C_1 \gg C_2$. The performance of the meta-atom was further optimized by finite-difference time-domain (FDTD) simulations. An incident SPP was launched onto an array of identical meta-atoms, and the geometric parameters were optimized to maximize the ratio between the RCP and LCP components. In particular, the spacing between the two nanobricks was optimized to improve the circular-polarization purity considering the practical strong coupling at the bottom of nanobricks, which ultimately led to the V-shaped meta-atom geometry. Supplementary Fig. 1b shows the simulated far-field intensity at 670 nm, in which LCP is magnified 5 times to see the pattern. The meta-atom produces highly directional circularly polarized emission, with RCP/LCP intensity ratio 125.4. The degree of circular polarization (DOCP) reaches as high as 98.4%, confirming the effectiveness of the design for high-purity spin-selective outcoupling.

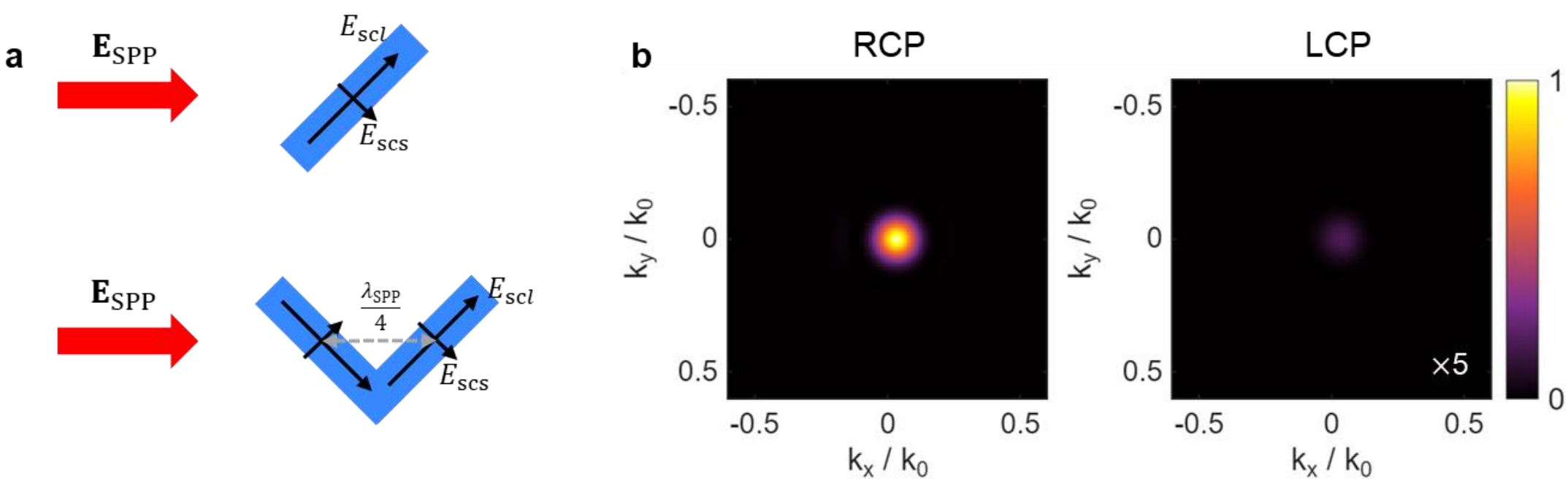

Supplementary Fig. 1 Meta-atom design for QE polarization control. **a,** Incident SPP scattered by dielectric anisotropic meta-atoms. Two cases are shown, where the meta-atom outcouples SPPs into free-space light with linear polarization (top) and circular polarization (bottom). **b,** Simulated far-field intensity distributions. The emission is dominated by the RCP channel, while the LCP component is significantly suppressed (scaled by a factor of 5), demonstrating efficient spin-selective scattering.

Supplementary Note 2 Generating skyrmionic beam

The simplest form of a skyrmionic beam can be constructed by superposing two orthogonally polarized LG modes with no radial nodes (p = 0), the same beam width, a common focal point, whose OAM differ by one, as follows[1]:

$$|\Psi(r)\rangle = \mathrm{LG}_0^0(r)|0\rangle + \exp(i\theta)\mathrm{LG}_0^1(r)|1\rangle \tag{4}$$

Here, $|0\rangle$ and $|1\rangle$ represent any two orthogonal optical polarization (e.g. $|\mathrm{R}\rangle$ and $|\mathrm{L}\rangle$), while $\mathrm{LG}_0^0(r)$ and $\mathrm{LG}_0^1(r)$ correspond to two orthogonal spatial modes. The global phase difference between the two modes is denoted by θ. In this case, the skyrmion number is $N_{\mathrm{sk}} = \Delta\ell = \ell_{\mathrm{L}} - \ell_{\mathrm{R}}$, which is the difference of topological charge of the two modes.

The normalized fundamental LG beams (p = 0), whose total intensity integrates to unity, are expressed at the beam waists ($z - z_0 = 0$, near field) as[2]:

$$\mathrm{LG}_0^{\ell}(r) = \left\| L_0^{\ell}(x) = 1 \right\| = \sqrt{\frac{2}{\pi|\ell|!}} \cdot \frac{1}{w} \cdot \exp\left(-\frac{r^2}{w^2}\right) \cdot \left(\sqrt{2}\frac{r}{w}\right)^{|\ell|} \cdot \exp(i\ell\varphi) \tag{5}$$

where $w = w_0$ is the beam waist, z denotes the propagation distance, r is the radial distance from the beam centre, φ is the azimuthal angle, and ℓ is the topological charge. The amplitude or intensity maximum occurs at $r_{max} = w\sqrt{\frac{|\ell|}{2}}$. The LG modes used to construct skyrmionic beams are therefore:

$$\mathrm{LG}_0^0 = \frac{\sqrt{2}}{w\sqrt{\pi}} \exp\left(-\frac{r^2}{w^2}\right) \tag{6}$$

$$\mathrm{LG}_0^{\pm 1} = \frac{\sqrt{2}}{w\sqrt{\pi}} \exp\left(-\frac{r^2}{w^2}\right) \cdot \left(\sqrt{2}\frac{r}{w}\right) \cdot \exp(\pm i\varphi) \tag{7}$$

By superposing these modes with opposite circular polarizations, a skyrmionic optical field can be written in the near field as:

$$|\boldsymbol{\Psi}(r, z=0)\rangle = \frac{\sqrt{2}}{w\sqrt{\pi}} \exp\left(-\frac{r^2}{w^2}\right) \left\{ \frac{1}{\sqrt{2}} \begin{pmatrix} 1 \\ -i \end{pmatrix} + \exp(i\theta) \cdot \left(\sqrt{2}\frac{r}{w}\right) \cdot \exp(i\varphi) \cdot \frac{1}{\sqrt{2}} \begin{pmatrix} 1 \\ i \end{pmatrix} \right\} =$$
$$= \frac{1}{w\sqrt{\pi}} \exp\left(-\frac{r^2}{w^2}\right) \left\{ \begin{pmatrix} 1 \\ -i \end{pmatrix} + \exp(i\theta) \cdot \left(\sqrt{2}\frac{r}{w}\right) \cdot \exp(i\varphi) \cdot \begin{pmatrix} 1 \\ i \end{pmatrix} \right\} \tag{8}$$

In the far-field ($z - z_0 \to \infty$), the fundamental LG mode is expressed as:

$$\mathrm{LG}_0^{\ell}(r, z \to \infty) = \sqrt{\frac{2}{\pi|\ell|!}} \cdot \frac{1}{\alpha_0 z} \cdot \exp\left(-\frac{\alpha^2}{\alpha_0^2}\right) \cdot \left(\sqrt{2}\frac{\alpha}{\alpha_0}\right)^{|\ell|} \cdot \exp(i\ell\varphi) \cdot \exp\left(-i\frac{\alpha^2 z}{\alpha_0 w_0}\right)$$
$$\cdot \exp\left[-i(|\ell| + 1)\frac{\pi}{2}\right] \tag{9}$$

where $\alpha = r/z$ is the diffraction angle and $\alpha_0 = \frac{\lambda}{\pi w_0}$ defines the beam divergence.

Supplementary Fig. 2 demonstrates the calculated far-field intensity profiles of a skyrmionic optical field. The interference between the RCP and LCP components gives rise to a spatially structured polarization field with skyrmionic topology. By varying the relative phase difference θ between the two modes, the optical field evolves continuously between Néel- and Bloch-type skyrmions characterized by different helicity parameters γ. Furthermore, more versatile forms of optical skyrmions could be realized by combining LG modes with different topological charges. When the topological charge difference $\Delta\ell$ between the two modes is varied, distinct skyrmionic textures emerge. For example, $\Delta\ell = +1$ and $\Delta\ell = -1$ enable generation of Néel-type and anti-type skyrmions, respectively. $\Delta\ell = -2$ leads to the formation of a high-order anti-type skyrmion featuring a multi-lobed polarization structure and enhanced topological complexity. These results demonstrate that the skyrmion number and helicity of optical skyrmions can be flexibly tailored by the two LG modes.

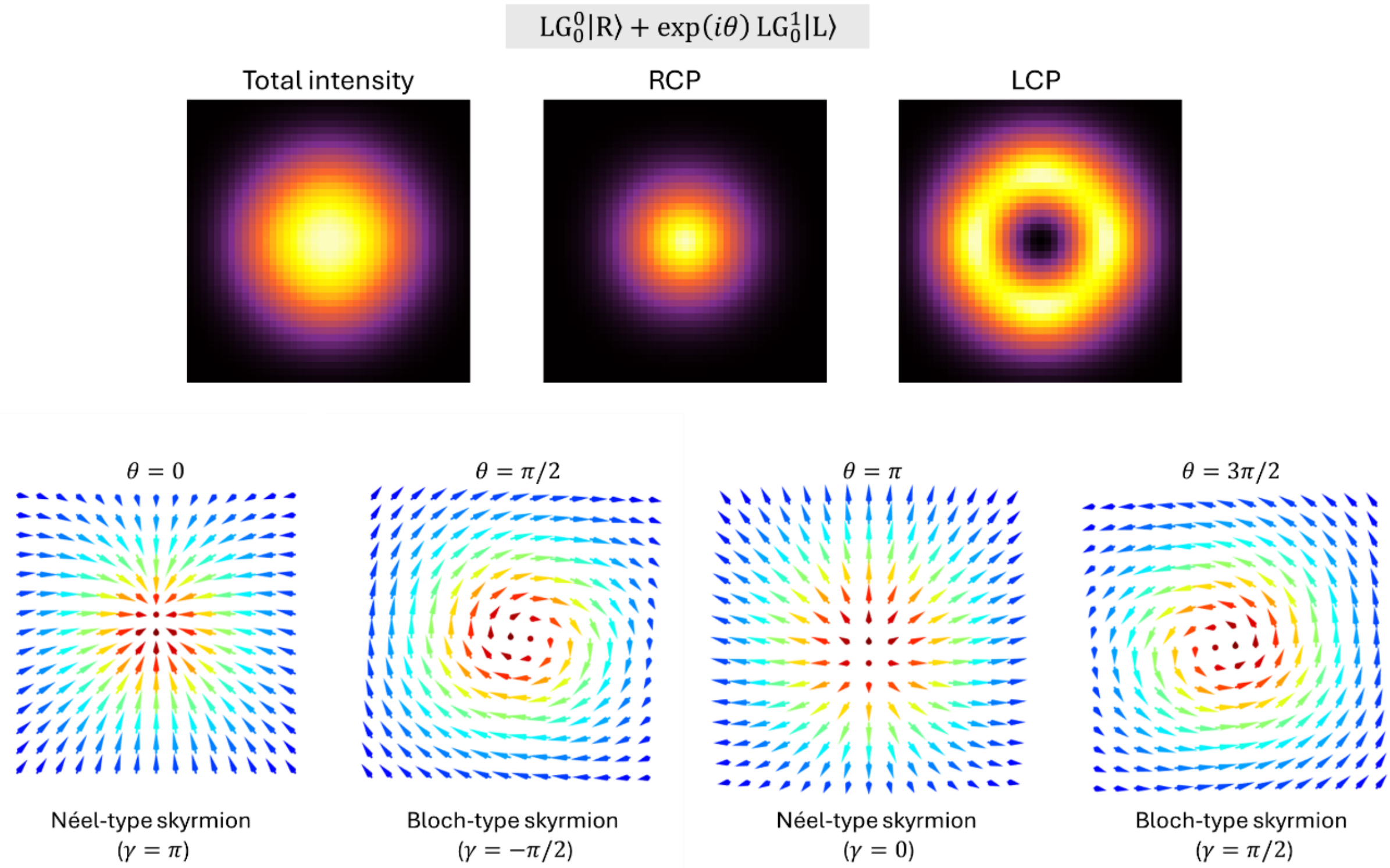

Supplementary Fig. 2 Far-field intensity profiles of a skyrmionic optical field composed of two LG modes. Tuning the azimuthal phase difference θ between the modes leads to the continuous evolution between Néel- and Bloch-type skyrmions characterized by different helicity parameters γ. The arrows indicate the local transverse polarization orientation, and the colour scale encodes the local polarization state, ranging from $S_3 = +1$ (red) to $S_3 = -1$ (blue).

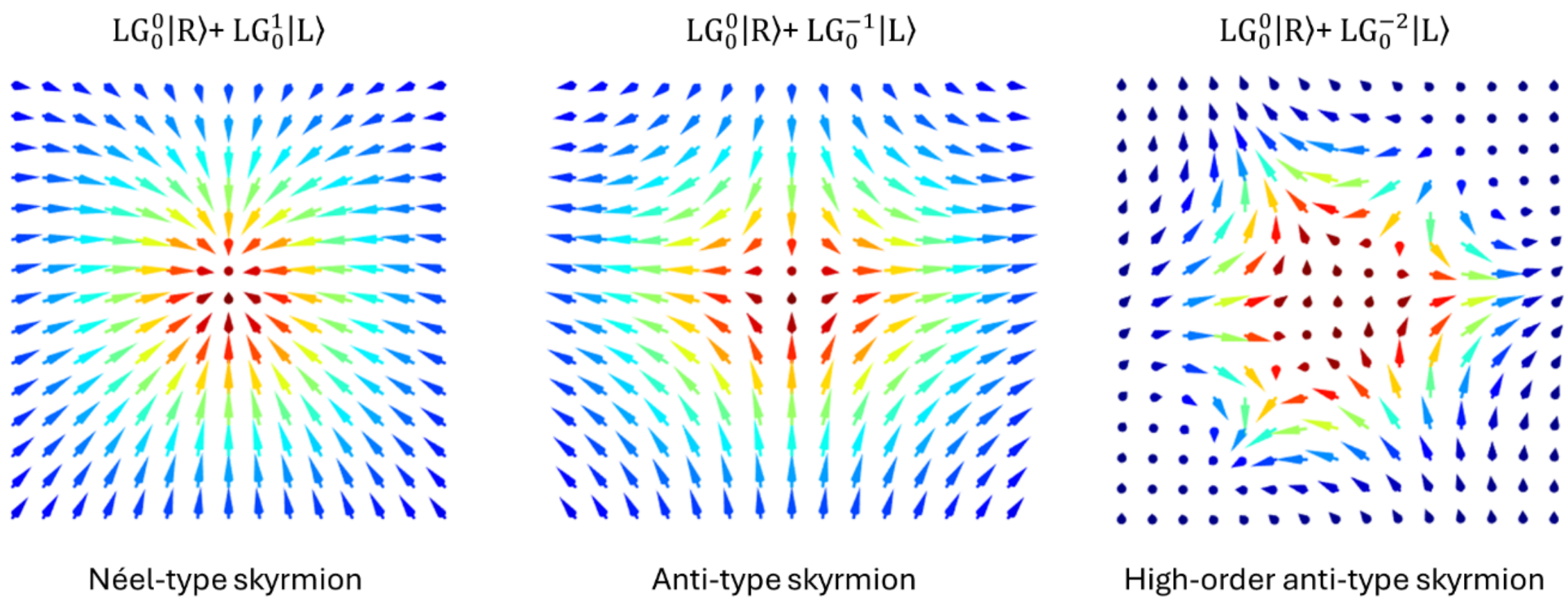

Supplementary Fig. 3 Polarization vector distributions of optical skyrmions formed by the interference between right- and left-circularly polarized LG modes with different topological charges.

For constructing skyrmionium beams, the fundamental LG beams with radial mode ($p = 1$) are used, which are described at their waists ($z - z_0 = 0$, near field) as follows:

$$\mathrm{LG}_1^{\ell}(r) = \sqrt{\frac{2}{\pi(1+|\ell|)!}} \cdot \frac{1}{w} \cdot \exp\left(-\frac{r^2}{w^2}\right) \cdot \left(\sqrt{2}\frac{r}{w}\right)^{|\ell|} \cdot \left(1 + |\ell| - \frac{2r^2}{w^2}\right) \exp(i\ell\varphi) \qquad (10)$$

where $w = w_0$ is the beam waist, the field amplitude changes sign at the radius $r_{cross} = w\sqrt{\frac{1+|\ell|}{2}}$. Here, different radial modes for RCP and LCP fields are used to avoid spatial overlap between them. Thus, the skyrmionium field can be constructed by superposing LG_0^1 and LG_1^{-1} modes with opposite circular polarizations:

$$|\boldsymbol{\Psi}(r)\rangle = \mathrm{LG}_0^1(r)|\mathrm{R}\rangle + \exp(i\theta)\,\mathrm{LG}_1^{-1}(r)|\mathrm{L}\rangle \qquad (11)$$

Substituting Eqs. (7) and (10) into Eq. (11) yields the following expression for the skyrmionium field in the near field:

$$|\boldsymbol{\Psi}(r, z = 0)\rangle = \frac{\sqrt{2}}{w\sqrt{\pi}} \exp\left(-\frac{r^2}{w^2}\right) \cdot \left(\sqrt{2}\frac{r}{w}\right) \cdot \exp(i\varphi) \cdot \frac{1}{\sqrt{2}}\begin{pmatrix}1\\-i\end{pmatrix} +$$

$$+ \exp(i\theta) \cdot \frac{1}{w\sqrt{\pi}} \exp\left(-\frac{r^2}{w^2}\right) \cdot \left(\sqrt{2}\frac{r}{w}\right) \cdot \left(2 - \frac{2r^2}{w^2}\right) \exp(-i\varphi) \cdot \frac{1}{\sqrt{2}}\begin{pmatrix}1\\i\end{pmatrix} =$$

$$= \frac{1}{w\sqrt{\pi}} \exp\left(-\frac{r^2}{w^2}\right) \cdot \left(\sqrt{2}\frac{r}{w}\right) \left\{\exp(i\varphi) \cdot \begin{pmatrix}1\\-i\end{pmatrix} + \exp(i\theta) \cdot \left(2 - \frac{2r^2}{w^2}\right) \cdot \exp(-i\varphi) \cdot \frac{1}{\sqrt{2}}\begin{pmatrix}1\\i\end{pmatrix}\right\} \qquad (12)$$

The top row of Supplementary Fig. 4 shows the calculated intensity distributions of the superposed field at $\theta = 0$. The corresponding decomposed components indicate the spatial separation of the RCP and LCP contributions. The resulting spatial polarization-vector distributions shown in the bottom row exhibit a nested polarization texture with double radial reversal, revealing a skyrmionium-like topology. As θ varies from $0, \pi/2$ to π, the skyrmionium field exhibits continuous rotation and internal twisting, corresponding to different helicity parameters ($\gamma = \pi, \pi/2, 0$).

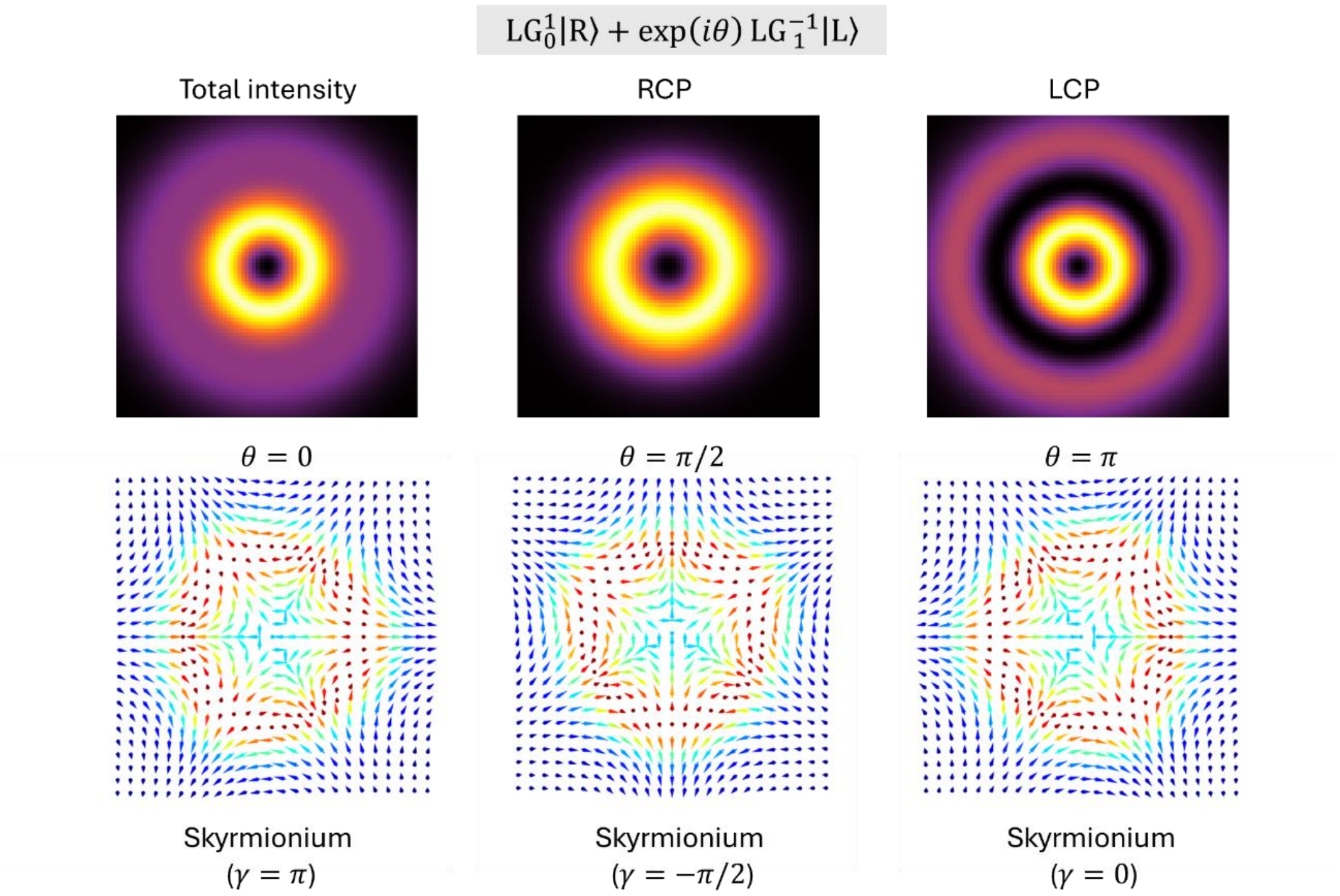

Supplementary Fig. 4 Skyrmionium polarization textures formed by the superposition of two spin–orbit modes. Top row: Calculated intensity distributions of the superposed field, along with the corresponding decomposed RCP and LCP components. Bottom row: Corresponding spatial polarization vector distributions, revealing a skyrmionium-like topology in real space. Varying the global phase offset θ rotates the skyrmionium texture while preserving its nontrivial topological character.

Supplementary Note 3 Device fabrication

The fabrication workflow of the metasurface-integrated quantum emitters is summarized in Supplementary Fig. 5. The fabrication process of metaQE is illustrated as follows. A 150 nm-thick Ag film was first thermally evaporated onto a silicon substrate, followed by deposition of a 20 nm SiO_2 layer using magnetron sputtering. Arrays of gold alignment markers were then defined on the substrate by electron-beam lithography (EBL, JEOL-6490, accelerating voltage 30 kV), gold deposition, and a standard lift-off process. A solution of nanodiamonds (NDs) containing colour centres was prepared at an optimized concentration and subsequently spin-coated onto the prepared substrates to obtain well-isolated NDs. The positions of the NDs were identified using dark-field microscopy images, with reference to the alignment markers. After determining the ND positions, a negative resist layer of hydrogen silsesquioxane (HSQ) was spin-coated at 2200 rpm for 45 s and prebaked at 160 °C for 2 min, forming a ~150 nm-thick layer verified by atomic force microscopy (NT-MDT NTEGRA). Metasurface structures were then carried out around the selected NV–NDs by EBL. The final metaQE devices were obtained after development in 25% tetramethylammonium hydroxide (TMAH) for 4 min, followed by rinsing in isopropanol for 60 s.

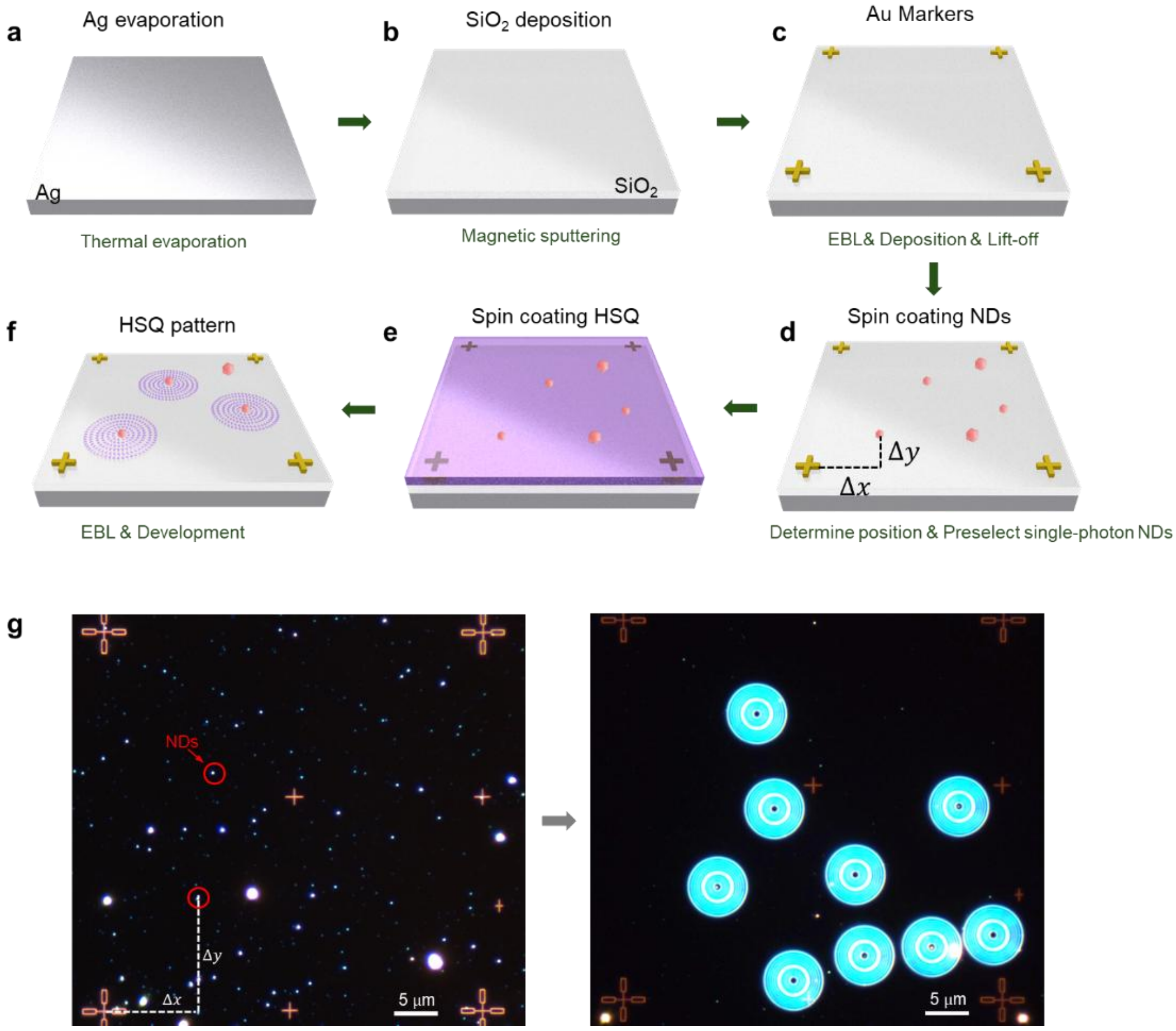

Supplementary Fig. 5 Schematic illustration of the fabrication process of meta-QE devices. **a,** Thermal evaporation of a 150-nm-thick Ag film onto a Si substrate. **b,** Deposition of a 20 nm SiO_2 layer by magnetron sputtering to form the metal–dielectric base. **c,** Fabrication of gold alignment markers by electron-beam lithography, gold deposition and lift-off. **d,** Spin-coating of nanodiamonds (NDs), followed by position identification with respect to the alignment markers. **e**, Spin-coating of a hydrogen silsesquioxane (HSQ) resist layer for electron-beam lithography patterning, followed by alignment of the metasurface pattern to the preselected NDs. **f,** Electron-beam lithography writing and development. **g,** Dark-field images of spin-coated NDs before and after coupling to the metasurface.

Supplementary Note 4 Optical Characterization

Optical measurements were performed using a confocal micro-photoluminescence setup, as shown in Supplementary Fig. 6. The NDs were optically excited by a radially polarized laser beam focused onto the sample through a 100×, NA = 0.9 objective lens. The fluorescence photon count rate was recorded using an avalanche photodiode (APD1) while scanning the sample with a piezoelectric stage, producing fluorescence maps for locating individual QEs. Polarization-resolved measurements were performed by inserting a broadband quarter-wave plate mounted on a motorized rotation stage and a linear polarizer into the detection path. A flip mirror was used to redirect the collected emission to a spatial light modulator (SLM) for characterization of the topological charge. The SLM was programmed with computer-generated phase holograms corresponding to different orbital angular momentum (OAM) modes. By projecting the emitted field onto these holograms and recording the first-order diffraction patterns on a CMOS2 camera, the topological charge of the structured emission was determined from the intensity distribution (Fig. 2f). Second-order correlation measurements were performed to confirm the single-photon emission. Second-order correlation measurements were carried out in a Hanbury Brown-Twiss (HBT) configuration to verify the quantum nature of the emitted photons (Supplementary Fig. 7). The collected fluorescence was split by a 50:50 beam splitter into two detection channels connected to avalanche photodiodes (APD1 and APD2). Photon arrival times were recorded by a time-correlated single-photon counting (TCSPC) module (PicoHarp 300), operating in a start-stop mode. The coincidence histogram of detection events as a function of the delay time τ yielded the $g^2(\tau)$ function, which was normalized to unity at long delays. A dip below 0.5 at $\tau = 0$ confirmed the single-photon emission from individual colour centres.

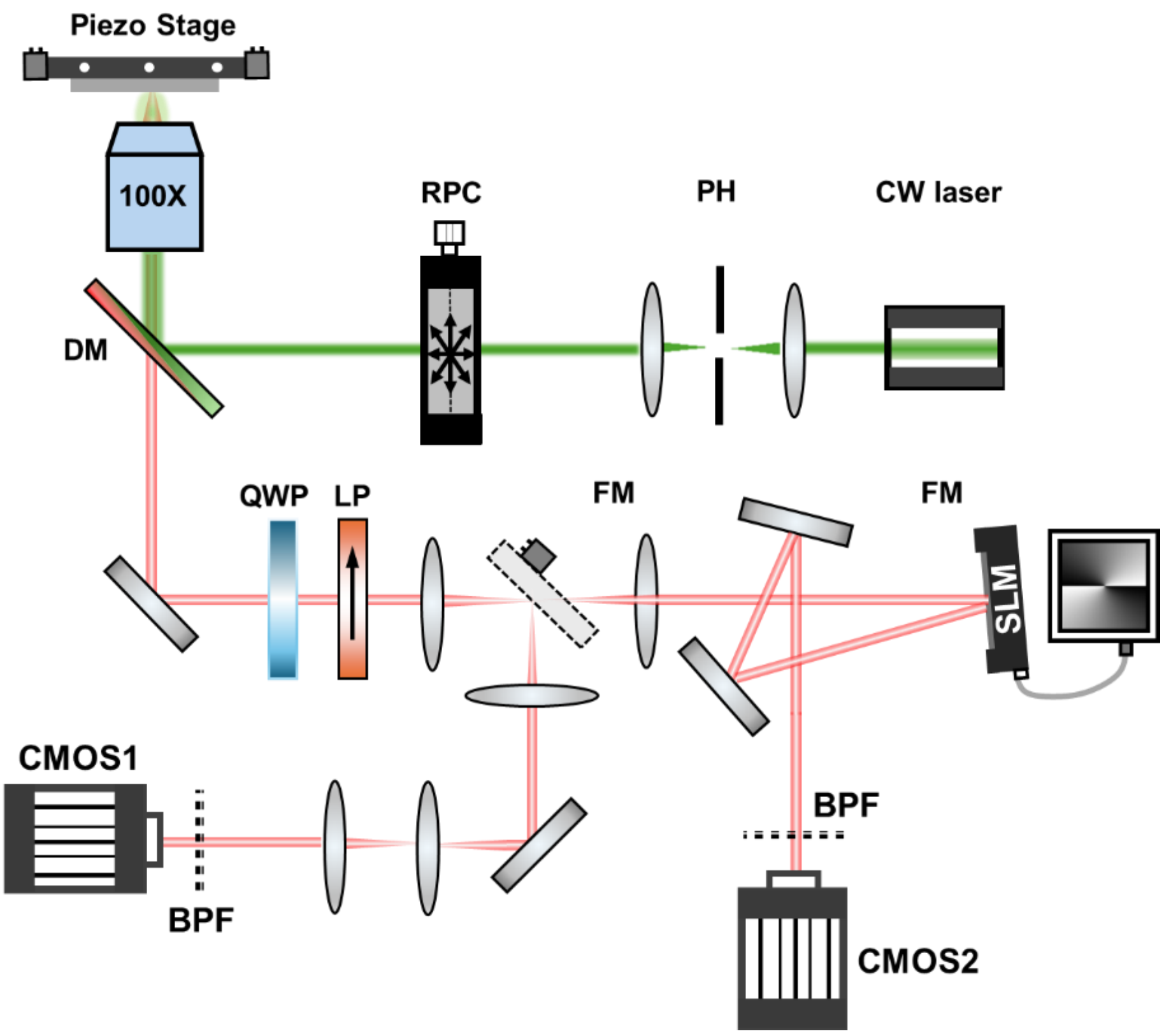

Supplementary Fig. 6 Schematic of the optical setup used for characterizing the meta-QE emission. The excitation beam is radially polarized and focused onto the sample through a 100×, NA = 0.9 objective. The spatially structured radiation pattern is captured at Fourier-plane images. CW: continuous wave, RP: Radial Polarization Converter, PBS: polarized beam splitter, PH: pinhole, DM: dichroic mirror, LP: linear polarization, QWP: quarter-wave plate, LPF: 550 nm long pass filter, FM: flip mirror.

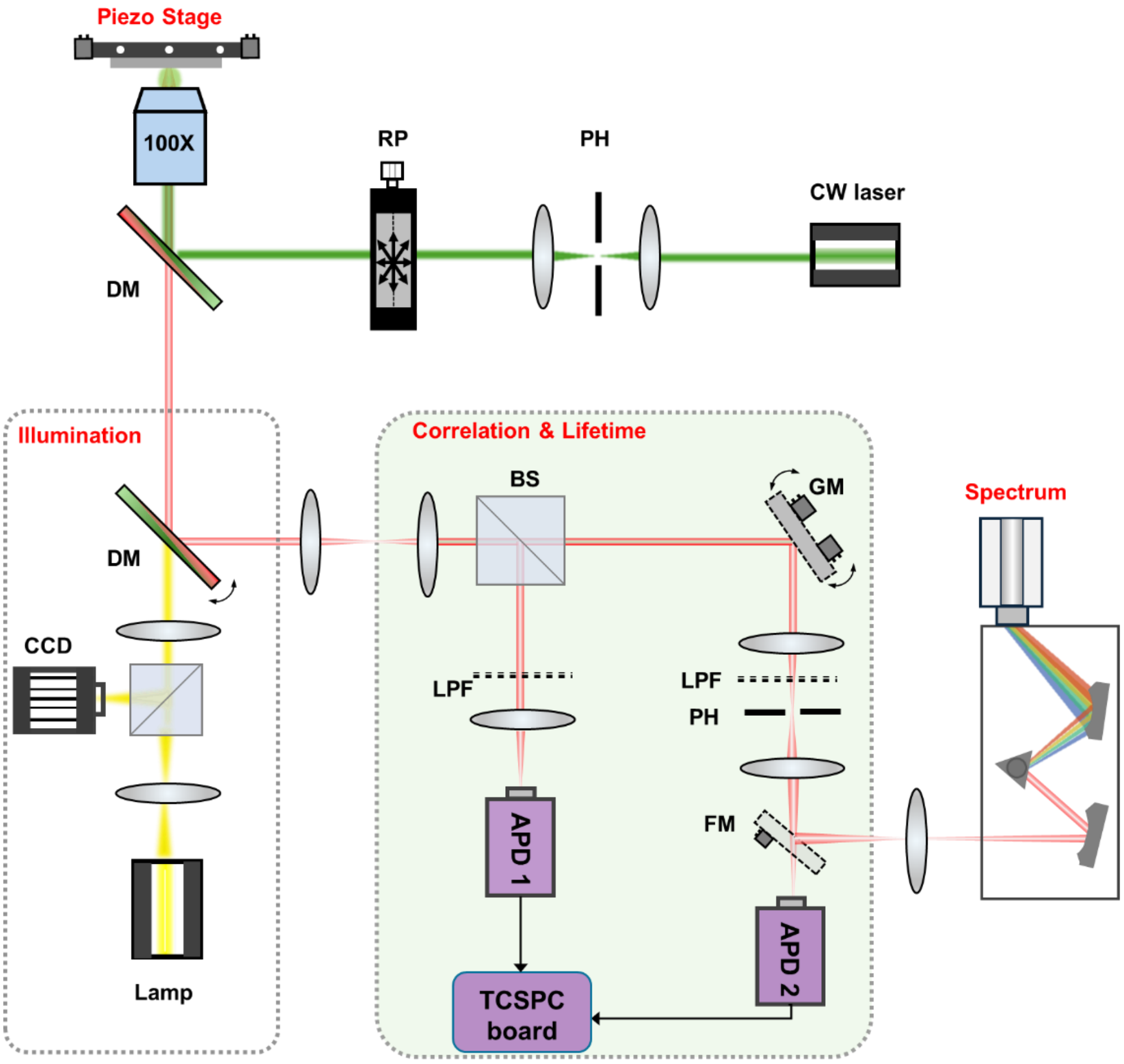

Supplementary Fig. 7 Experimental setup for optical characterization for correlation, lifetime, and spectral measurements. GM: galvanometric mirror. APD: Avalanche Photodiode.

Supplementary Note 5 Measurement of spatial polarization states and polarization ellipse

The Stokes vector $\mathbf{S} = [S_0, S_1, S_2, S_3]$ is used to describe the degree of polarization for any state of light. S_0 represents the total intensity, S_1 and S_2 quantify the degree of linear polarization, and S_3 describes the degree of circular polarization. For a plane wave with orthogonal components $\boldsymbol{E_x} = A_x e^{i\varphi_x}$, $\boldsymbol{E_y} = A_y e^{i\varphi_y}$. The Stokes parameters are defined as:

$$
\begin{aligned}
S_0 &= \boldsymbol{E_x}\boldsymbol{E_x^*} + \boldsymbol{E_y}\boldsymbol{E_y^*} = |A_x|^2 + |A_y|^2 \\
S_1 &= \boldsymbol{E_x}\boldsymbol{E_x^*} - \boldsymbol{E_y}\boldsymbol{E_y^*} = |A_x|^2 - |A_y|^2 \\
S_2 &= \boldsymbol{E_x}\boldsymbol{E_y^*} + \boldsymbol{E_y}\boldsymbol{E_x^*} = 2|A_x||A_y|\cos\delta \\
S_3 &= i(\boldsymbol{E_x}\boldsymbol{E_y^*} - \boldsymbol{E_y}\boldsymbol{E_x^*}) = 2|A_x||A_y|\sin\delta
\end{aligned} \tag{13}
$$

where $\boldsymbol{E_x^*}$ and $\boldsymbol{E_y^*}$ are the complex conjugates of $\boldsymbol{E_x}$ and $\boldsymbol{E_y}$, respectively, and where $\delta = \varphi_y - \varphi_x$ is the phase difference between the two field components.

To experimentally characterize the spatially varying polarization states of the emitted photons, we performed full Stokes polarimetry based on intensity quantities. The emitted photons pass through a quarter wave plate (retardation angle δ). Then the beam passes a linear polarizer with its transmission axis aligned at an angle α to the *x*- axis. The far-field image was recorded for four combinations of δ and α to calculate the four Stokes parameters. The first three Stokes parameters are measured by rotating the linear polarizer to angle $\alpha = 0°,\ 90°,\ 45°, \text{and} - 45°$ respectively (without the quarter wave plate). The final parameter S_3 is measured using linear polarizer and the quarter wave retarder ($\alpha = \pm 45°, \delta = 90°$). The Stokes parameters are derived as:

$$
\begin{aligned}
S_0 &= I(0°, 0°) + I(90°, 0°) \\
S_1 &= I(0°, 0°) - I(90°, 0°) \\
S_2 &= I(45°, 0°) - I(-45°, 0°) \\
S_3 &= I(45°, 90°) - I(-45°, 90°)
\end{aligned} \tag{14}
$$

The normalized Stokes parameters $\mathbf{s} = [S_0, S_1, S_2, S_3]/S_0$ were subsequently employed to calculate the local polarization ellipse. The shape and handedness of the ellipse are characterized by the ellipticity angle χ and the orientation angle ψ, which can be derived from the normalized Stokes parameters as

$$\sin 2\chi = S_3;\ \tan 2\psi = \frac{S_2}{S_1} \tag{15}$$

Here, ψ describes the rotation of the polarization ellipse with respect to x-axis, and χ quantifies its ellipticity, where $\chi = 0°$ corresponds to linear polarization and $\chi = \pm 45°$ corresponds to right or lefthanded circular polarization. By mapping ψ and χ across the beam profile, the local polarization ellipses can be reconstructed, providing an intuitive visualization of the spatially varying polarization states and their topological organization in skyrmionic and skyrmionium beams. The reconstructed polarization ellipses provide a direct visualization of the spatial polarization topology. To quantify the topological nature of the polarization field, we calculate the skyrmion number N_{sk}, which represents

the number of times the normalized Stokes vector wraps around the Poincaré sphere. A nonzero N_{sk} indicates a topologically nontrivial polarization configuration. For instance, a value close to +1 or -1 corresponds to a skyrmion or antiskyrmion, while a near-zero value with an internal spin reversal indicates a skyrmionium structure.

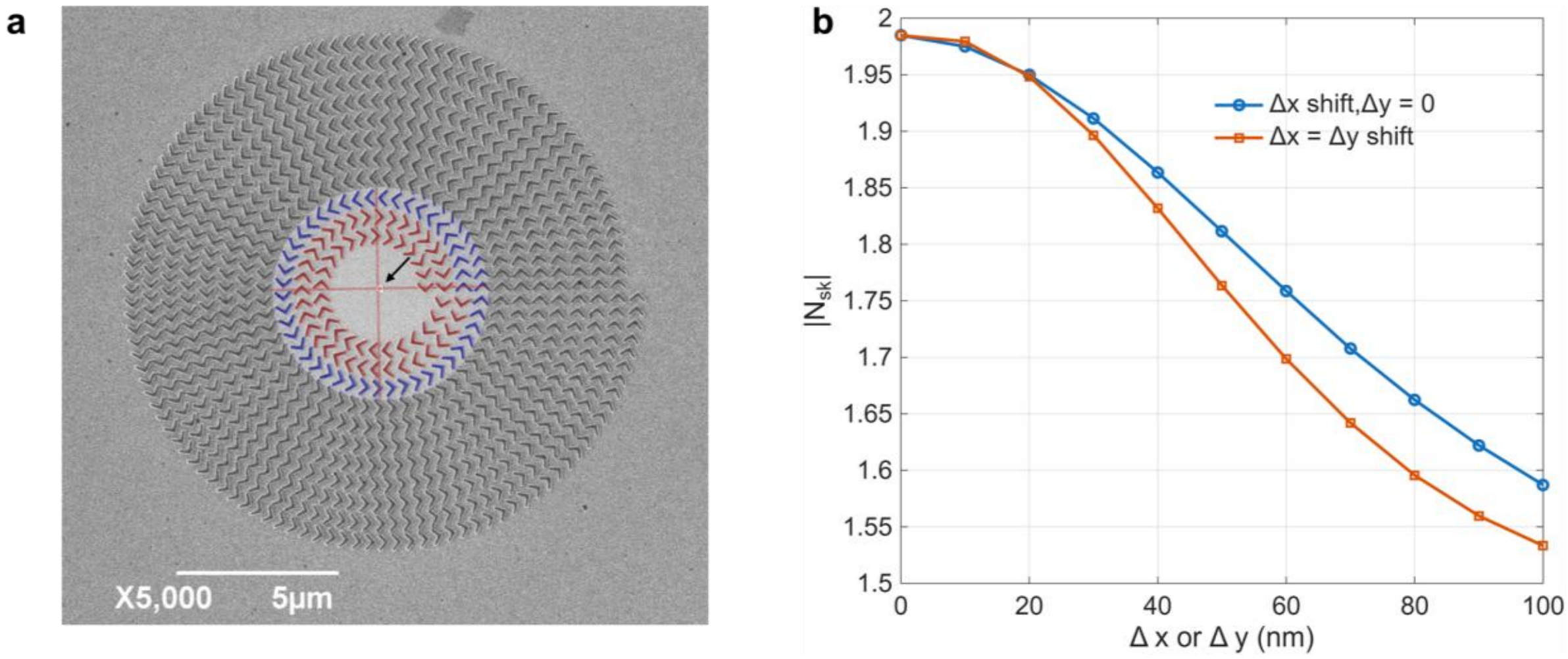

Supplementary Fig. 8 Structural characterization and tolerance to QE misalignment. **a,** Scanning electron microscope (SEM) image of the fabricated metasurface. The central region is overlaid with the corresponding design layout to estimate the QE position. **b,** Calculated skyrmion number as a function of QE positional misalignment. Two cases are considered: displacement along the x direction (Δx, Δy = 0) and equal displacements along both x and y directions (Δx = Δy).

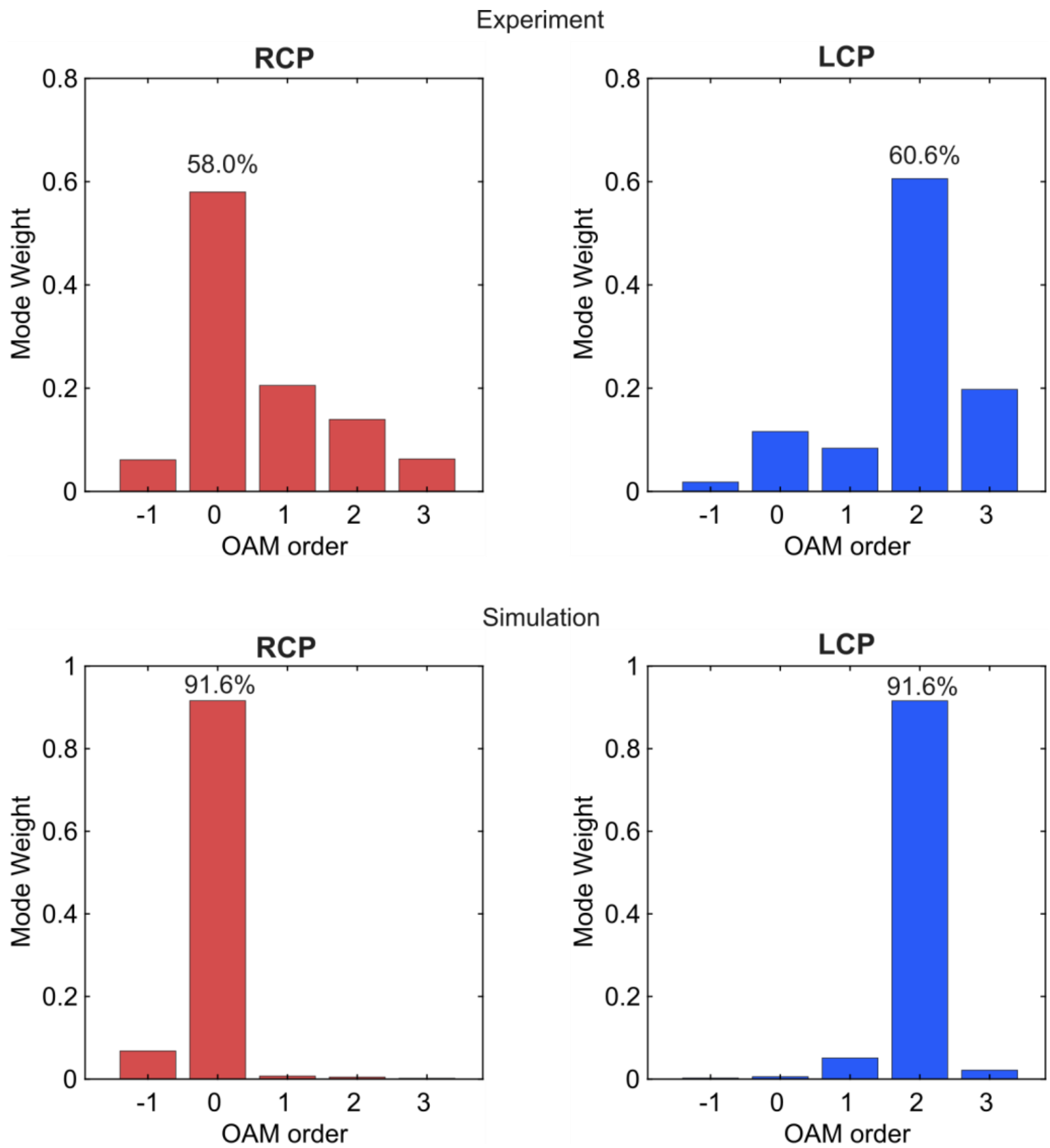

Supplementary Fig. 9 Measured and simulated OAM mode distributions under circular polarization bases. The distributions were obtained by projecting the emitted photons onto different LG_0^ℓ modes using a spatial light modulator (SLM), and the corresponding intensities were recorded. The x-axis denotes the OAM mode encoded on the SLM. Both experiment and simulation show dominant contributions at $\ell = 0$ for RCP and $\ell = 2$ for LCP.

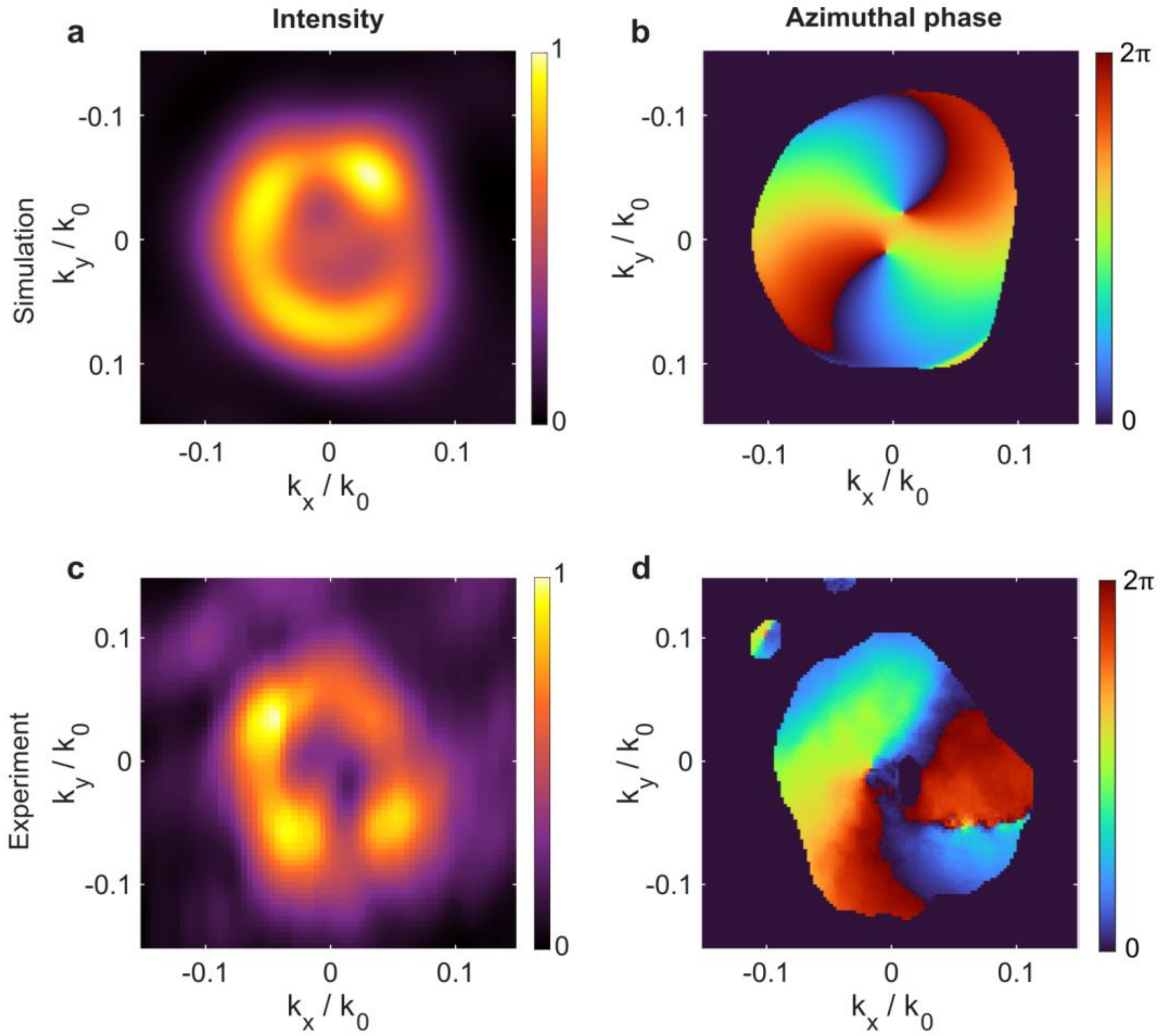

Supplementary Fig. 10 Simulated and experimental far-field intensity and spatially resolved (relative) phase distributions. **a, c,** Simulated and measured far-field intensity distributions in momentum space. **b, d,** Corresponding spatially resolved phase distributions between the two spin–orbit components, retrieved from the Stokes parameters according to $\phi = \mathrm{atan2}(S_2, S_1) + \pi$ (following the method used in Ref[3]), showing a 4π phase winding in the azimuthal direction.

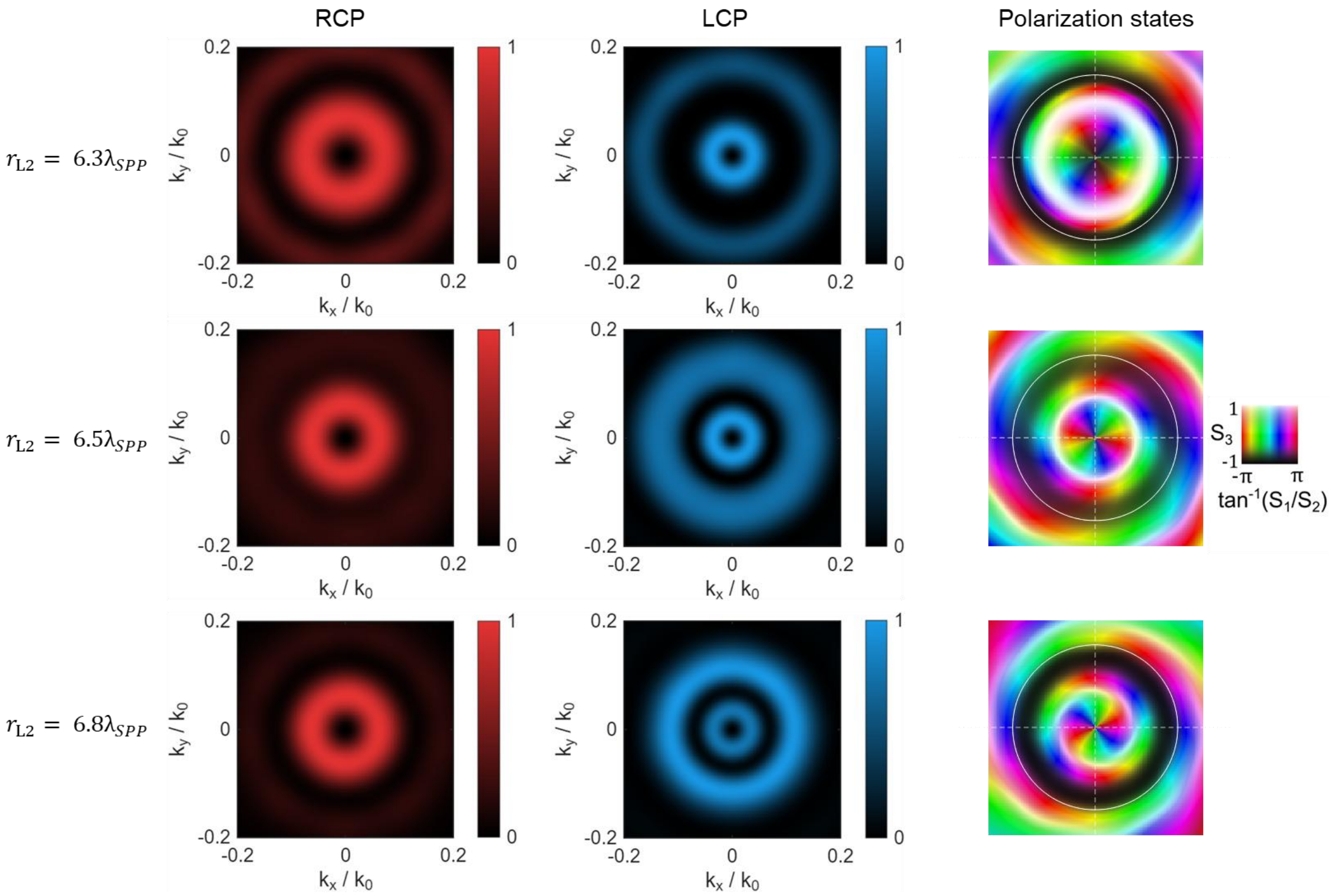

Supplementary Fig. 11 Simulated far-field distributions of photon emissions for three metasurface configurations with different initial radii r_{L2}. From left to right: intensity of the RCP and LCP components, the Stokes parameter S_3, and the spatial polarization state distribution. The sequential variation in the velocity of the polarization field verifies the tunable skyrmionium-like topology governed by the metasurface design, where increasing the initial radius of the meta-atoms systematically modifies the azimuthal phase accumulation.

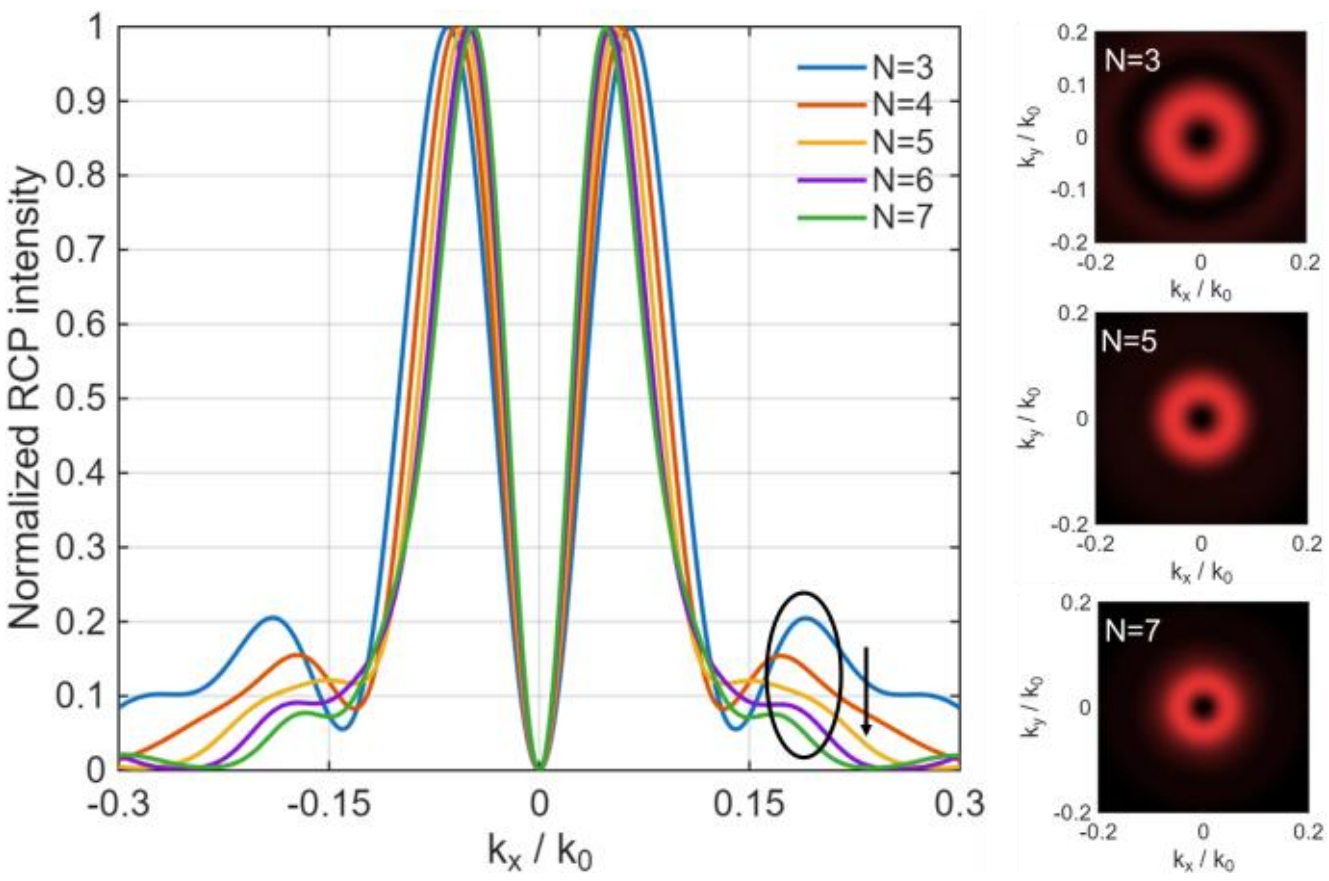

Supplementary Fig. 12 Simulated RCP far-field intensity distributions of metasurfaces composed only of RCP units for different ring numbers N. The weak outer side lobe gradually decreases with increasing N. The right column shows the corresponding two-dimensional far-field intensity distributions for $N = 3$, 5, and 7.

Supplementary References

1. Gao, S. *et al.* Paraxial skyrmionic beams. *Phys. Rev. A* **102**, 053513 (2020).
2. Nape, I., Sephton, B., Ornelas, P., Moodley, C. & Forbes, A. Quantum structured light in high dimensions. *APL Photonics* **8**, 051101 (2023).
3. Zhang, Z. *et al.* Spin–orbit microlaser emitting in a four-dimensional Hilbert space. *Nature* **612**, 246–251 (2022).
