# Supplementary material for: Chip-integrated metasurface-enabled single-photon skyrmion sources
Source: Nat Commun. 2026 Jul 11;17:8567. doi: 10.1038/s41467-026-75516-5 (PMC13483359; doi:10.1038/s41467-026-75516-5)
Supplement: Supplementary file 2 [file 41467_2026_75516_MOESM2_ESM.pdf]

nature portfolio

Peer Review File

Chip-integrated metasurface-enabled single-photon skyrmion sources

Corresponding Author: Professor Yinhui Kan

This file contains all reviewer reports in order by version, followed by all author rebuttals in order by version.

Version 0:

Reviewer comments:

Reviewer #1

(Remarks to the Author)
This manuscript reports the first on-chip generation of single-photon skyrmions—including high-order anti-skyrmions and skyrmionium—at room temperature, achieved through a metasurface-integrated quantum emitter (metaQE) platform. This work successfully extends photonic topology into the single-photon regime, adopting a rigorous technical methodology characterized by the tight integration of experimental investigations and simulations, along with sufficient data to robustly support its core conclusions. The metaQE platform demonstrated herein exhibits excellent universality and scalability, thereby laying a critical foundation for the development of scalable quantum photonic devices and high-dimensional quantum information processing. Accordingly, I recommend the acceptance of this manuscript following revisions aimed at clarifying certain definitions and theoretical details, as well as situating portions of the experimental data.
My specific comments are detailed below:
1) Extensive research has already been conducted on single-photon skyrmion sources and the mediation of spin-orbit interactions by meta-atoms. The authors should better situate their work within the framework of these prior studies, several of which are referenced below.
a) The reference cited by the authors (Ref. 30) has already realized the effective generation of single-photon skyrmions. While the authors have highlighted the advantages of their own work—including room-temperature operation and no requirement for an external magnetic field—they should conduct a more detailed comparison with this reference to deepen readers' understanding of their unique contributions.
b) Multiple studies have reported the manipulation of photonic spin-orbit interactions using metasurfaces, including control over both angular (Science 358, 896, 2017) and radial (Physical Review Applied 15, 014059, 2021) degrees of freedom. The authors should elaborate on how their work differs from these previous demonstrations and how it builds upon the existing body of knowledge.
2) The authors have established a theoretical framework for generating tunable skyrmions using metasurfaces; however, further clarifications are required for the following points:
a) The authors' previous work has demonstrated that concentric rings can modulate surface plasmon polaritons. It is recommended that the authors explain in the supplementary materials why L-shaped meta-atoms oriented at ±45° can modulate surface plasmon polaritons with different polarization states, with the explanation tailored to the comprehension level of non-specialist readers.
b) A mathematical expression (e.g., analogous to Eq. 1 for skyrmions) is lacking for skyrmionium. Although the authors provided the expression for the Laguerre-Gaussian (LG) superposition state in the supplementary materials, the decomposition results for skyrmionium were not presented. The authors should supplement this key theoretical content to ensure the completeness of their framework.
3) The experimental data presented by the authors provide compelling evidence for the effective generation of single-photon skyrmions; nevertheless, certain details need to be supplemented to enhance the rigor and completeness of the results:
a) In Figure 2, the authors demonstrate the coupling of different orbital angular momentum (OAM) states with distinct polarization states. To better illustrate the deviation between the experimental and theoretical values of N, the authors should provide the OAM spectral distribution in the measured OAM space. Additionally, phase is a crucial parameter governing skyrmion types; thus, the authors should measure the relative phase and its associated uncertainty. This data can be obtained via projection measurements of the polarization and OAM degrees of freedom using the experimental setup described in the supplementary materials.
b) Similarly, the relative error for $g^2(0)$ was not provided. The authors should supplement this error analysis to ensure the reliability and reproducibility of the experimental results.

(Remarks on code availability)

Reviewer #2

(Remarks to the Author)
In this manuscript Liu et. al propose and implement a metasurface coupled with quantum emitters (QEs) to create single-photon emission with skyrmionic polarization states. Optical skyrmions are, in general, a rich playground for topological photonics. Creation of various skyrmions has seen enormous effort in a variety of systems, including photonics, acoustics and magnetic systems. For free-space optical skyrmions, the first key step for such a system is to engineer distinct orbital angular momentum (OAM) modes with orthogonal polarizations. The authors employ a metasurface composed of two distinct types of meta-atoms, designed to generate individual Laguerre-Gaussian (LG) modes with right circular polarization (RCP) and left circular polarization (LCP), respectively. By superposing these two modes and coupling them to QEs, the emission states of the single photons can be controlled to obtain skyrmionic polarization distributions.

While I think the sample fabrication and QE implementation in this work are technically sophisticated, the authors don't convincingly demonstrate conceptual and theoretical novelty. For these reasons I cannot recommend this manuscript for publication as I found it incremental and does not meet the high standard of Nature Communications. I describe my reservations in more detail below.

First, a note on the generation of arbitrary OAM modes and polarizations using metasurfaces: There are tremendous design strategies for optical metasurfaces that can generate various modes of OAMs and polarizations, including the LG modes with RCP/LCP polarizations. In this work, the key to generating a single-photon emission with a skyrmionic polarization distribution is the precise excitation of specific LG modes, the requirements for which are well established in Ref. [29] cited by the authors.

Second, a note on the implementation of QEs in the color center of a metasurface:
I found very similar works reported by some of the authors of the current manuscript demonstrating respectively:
A) (as in Ref. [32], Kan, Y. et al. Adv. Mater. 32, 1907832 (2020)) dipolar QEs placed in the color center and coupled to surface plasmon polariton (SPP) modes of a metasurface composed of circular nanoridges, which upon interaction with an optical metasurface radiate into free space in the form of a collimated singlephoton stream carrying the designed chirality, i.e., RCP or LCP;

B) (as in Ref. [26], Kan, Y. et al. Sci. Adv. 10, eadq6298 (2024)) QEs placed in the color center of Moiré metasurfaces enable the generation of structured single photon emission vortex beams with vectorial spin-orbital states. Similarly, the control of the emission state is through coupling the QEs with RCP and LCP, each carrying two different OAMs.

Therefore, the combination of these two concepts - metasurface-based LG mode generation and QE-excited SPPs appears to be an incremental development, which may not meet the high bar of scientific significance set by Nature Communications, although the presentation is technically sound and the correctness of the results is unquestionable.

(Remarks on code availability)

Reviewer #3

(Remarks to the Author)
I co-reviewed this manuscript with one of the reviewers who provided the listed reports. This is part of the Nature Communications initiative to facilitate training in peer review and to provide appropriate recognition for Early Career Researchers who co-review manuscripts.

(Remarks on code availability)

Reviewer #4

(Remarks to the Author)
In this manuscript, the authors report room temperature, on-chip generation of single-photon skyrmions using a metasurface-integrated quantum emitter platform. By engineering arrays of subwavelength meta-atoms, the authors shape the emission from different colour centres in nanodiamonds into diverse skyrmionic single-photon states via spin-orbit interaction.
Overall, the work is interesting and timely. The fabrication, optical characterization, and experimental control appear technically sound. However, there are technical issues preventing its publication in the current form.
1. The manuscript emphasizes "on-chip generation, however, the Introduction would benefit from a more explicit discussion of recent integrated optical skyrmion source demonstrations (e.g., micro-ring(Optica, 11, 1588(2024)), q-plate(Phys. Rev.

Lett., 134, 083802 (2025)), bullseye cavity(Nanophotonics, 14(24), 4411 (2025), metasurface(Nanophotonics, 14(23), 4069 (2025))).
2. In lines 39-40, the authors state that “current implementations typically rely on complex optical setups, or require strict operational conditions, which hinder scalability and tunability”. However, the cited references (ref. 20, 27-29) at that location appear not fully aligned with the single-photon skyrmion context.
3. The authors state that the metasurface was “precisely integrated” with a nanodiamond containing NV centres (lines 109-112). Please provide a quantitative estimate of the nanodiamond positioning accuracy, and discuss how misalignment affects the quality of the emitted single-photon skyrmions.
4. The skyrmion number is defined in Eq.(2) as a 2D integral over x,y, but the integration domain is not specified in Eq.(2). In addition, the authors should explain how the skyrmion regions in Fig.3(d,f) and Fig.4(c,d) are determined.
5. The presentation of the results in Fig. 2 is somewhat confusing. (i) Please clarify whether Fig.2(c) shows simulated or experimental data. (ii) The manuscript reports a measured skyrmion number of -1.86, but it is not clearly stated which measurement this value is extracted from (presumably Fig.2(e)). In addition, the ideal value is -2. Please provide a quantitative analysis of the discrepancy.
6. In Fig.3(c,e), the authors aim to generate LG modes where the RCP component is described as having no radial mode. However, an additional ring-like feature appears in the measured intensity distribution. This extra ring could potentially introduce an extra polarization flip along the radial direction. Please explain this feature.
7. In Fig.4(d), the measured S3 distribution deviates from the expected circular symmetry and exhibits a clear two-lobed pattern. This could distort the skyrmion texture and thus influence the extracted skyrmion number. Please comment on the origin of this symmetry breaking and quantify its impact on the reconstructed skyrmion texture and the calculated skyrmion number.
8. The reported g2(0) 0.19 lacks uncertainty estimates.
9. In several figures, the colorbars indicate numerical ranges, but the underlying physical quantity represented by the color scale is not specified. In addition, some of these panels lack clear coordinate axes, making it difficult to interpret the spatial scale. This issue appears in Fig.2(c-f), Fig.3(c-f), and Fig.4(c-f).

(Remarks on code availability)

Reviewer #5

(Remarks to the Author)
I co-reviewed this manuscript with one of the reviewers who provided the listed reports. This is part of the Nature Communications initiative to facilitate training in peer review and to provide appropriate recognition for Early Career Researchers who co-review manuscripts.

(Remarks on code availability)

Version 1:

Reviewer comments:

Reviewer #1

(Remarks to the Author)
The authors have adequately addressed the reviewers' questions and revised the manuscript according to the reviewers' comments. This work combines metasurfaces with quantum dots to generate quantum skyrmions, which is of great significance for integrated quantum light sources. I recommend acceptance and publication.

(Remarks on code availability)

Reviewer #2

(Remarks to the Author)
The authors have provided clear answers to the questions raised.
The revised manuscript is now ready for publication.

(Remarks on code availability)

Reviewer #3

(Remarks to the Author)
I co-reviewed this manuscript with one of the reviewers who provided the listed reports. This is part of the Nature Communications initiative to facilitate training in peer review and to provide appropriate recognition for Early Career Researchers who co-review manuscripts.

(Remarks on code availability)

Reviewer #4

(Remarks to the Author)
I thank the authors for carefully addressing my comments. I have no more technical concerns.

However, I do agree with Reviewer 2, who brought my attention to many of the authors' papers published in recent years, which are all conceptually and technically similar to the results reported here.

Now, I am not sure whether the novelty of this work is sufficient for publication in Nature Communications.

(Remarks on code availability)

Reviewer #5

(Remarks to the Author)
I co-reviewed this manuscript with one of the reviewers who provided the listed reports. This is part of the Nature Communications initiative to facilitate training in peer review and to provide appropriate recognition for Early Career Researchers who co-review manuscripts.

(Remarks on code availability)

Open Access This Peer Review File is licensed under a Creative Commons Attribution 4.0 International License, which permits use, sharing, adaptation, distribution and reproduction in any medium or format, as long as you give appropriate credit to the original author(s) and the source, provide a link to the Creative Commons license, and indicate if changes were made.
In cases where reviewers are anonymous, credit should be given to 'Anonymous Referee' and the source.
The images or other third party material in this Peer Review File are included in the article's Creative Commons license, unless indicated otherwise in a credit line to the material. If material is not included in the article's Creative Commons license and your intended use is not permitted by statutory regulation or exceeds the permitted use, you will need to obtain permission directly from the copyright holder.
To view a copy of this license, visit https://creativecommons.org/licenses/by/4.0/

Response to reviewer's comments

(Listing all changes made)

First, we would like to thank the reviewers for their efforts in assessing our manuscript and very valuable comments. We have carefully considered each comment (highlighted below in blue) and provided detailed responses. Additionally, we have made the necessary revisions, which are highlighted in red in the manuscript. We consider the revised manuscript to be substantially improved and hope it now meets the criteria for publication. Below, we provide the point-by-point response to each reviewer's comment, along with the summary of all changes made.

Reviewer #1

This manuscript reports the first on-chip generation of single-photon skyrmions—including high-order anti-skyrmions and skyrmionium—at room temperature, achieved through a metasurface-integrated quantum emitter (metaQE) platform. This work successfully extends photonic topology into the single-photon regime, adopting a rigorous technical methodology characterized by the tight integration of experimental investigations and simulations, along with sufficient data to robustly support its core conclusions. The metaQE platform demonstrated herein exhibits excellent universality and scalability, thereby laying a critical foundation for the development of scalable quantum photonic devices and high-dimensional quantum information processing. Accordingly, I recommend the acceptance of this manuscript following revisions aimed at clarifying certain definitions and theoretical details, as well as situating portions of the experimental data.

Our response:

We appreciate the reviewer's overall positive evaluation and thoughtful summary of our work, noting its novelty, significance, and impact.

My specific comments are detailed below:

1) Extensive research has already been conducted on single-photon skyrmion sources and the mediation of spin-orbit interactions by meta-atoms. The authors should better situate their work within the framework of these prior studies, several of which are referenced below.

a) The reference cited by the authors (Ref. 30) has already realized the effective generation of single-photon skyrmions. While the authors have highlighted the advantages of their own work—including room-temperature operation and no requirement for an external magnetic field—they should conduct a more detailed comparison with this reference to deepen readers' understanding of their unique contributions.

b) Multiple studies have reported the manipulation of photonic spin-orbit interactions using metasurfaces, including control over both angular (Science 358, 896, 2017) and radial (Physical Review Applied 15, 014059, 2021) degrees of freedom. The authors should elaborate on how their work differs from these previous demonstrations and how it builds upon the existing body of knowledge.

Our response:

We thank the reviewer for these important remarks and would like to elaborate as follows:

First, regarding Ref. [30] (now Ref. [31]), we fully acknowledge that this is an important prior work demonstrating the generation of single-photon skyrmions using a quantum dot coupled to a Gaussian microcavity. Our work differs from Ref. [31] in both physical mechanism and practical operating regime. In Ref. [31], the skyrmionic emission relies on cavity-engineered modes and selective excitation enabled by Zeeman splitting, requiring both low temperature and an external magnetic field. In contrast, our approach is based on a chip-integrated metasurface-QE platform, in which QE-excited surface plasmon polaritons (SPPs) act as an intermediate channel between the nanoscale emitter and the metasurface. This mechanism allows us to shape the polarization and phase of the emitted single-photon field directly on chip, without requiring magnetic-field tuning or cryogenic operation. In addition, the freedom to design individual metasurface elements, which convert QE-excited propagating SPPs into free-propagating waves, enables more flexible generation of complex topological states, including not only anti-skyrmions but also high-order anti-skyrmions and skyrmionium.

Second, regarding previous metasurface-based demonstrations of photonic spin-orbit manipulation, including control over angular and radial degrees of freedom in free-space optical fields (Science 358, 896, 2017; Physical Review Applied 15, 014059, 2021), their primary focus was the structuring of externally incident classical light. By contrast, our work addresses a different regime and goal: the direct structuring of quantum emission originating from a nanoscale QE. In our platform, the QE first launches propagating surface excitations, SPPs, and then the metasurface converts these QE-excited SPPs into free-space single-photon states with designed spin-orbit textures. Therefore, the key advance here is not the spin-orbit control by metasurfaces *per se*, but its implementation in an on-chip QE-integrated quantum-emission platform operating at room temperature.

To clarify this in the manuscript, we have added the following discussion:

"…Upon excitation, the QE generates an electromagnetic dipole primarily polarized normal to the surface, that couples efficiently to nonradiative SPPs propagating radially along the dielectric-metal interface[35]. Here, QE-excited SPPs act as an intermediate channel between the nanoscale emitter and the metasurface, providing exceptional freedom for at-source tailoring spatially distributed phase and polarization of quantum emission. This operating principle is fundamentally different from previous metasurface studies based on externally incident free-space light[36,37]. With properly designed individual subwavelength-sized metasurface elements, meta-atoms, the QE-excited SPPs are outcoupled to free-space radiation with engineered spin-orbit textures, thereby generating the desired skyrmionic single-photon emission (Fig. 1a)." – page 2

"…In this way, both the azimuthal and radial mode orders of the emitted photons are controlled by the metasurface configuration, forming different LG modes with orthogonal polarizations. Consequently, the metaQE converts the nm-localized QE excitation into a free-space single-photon emission with spatially varying polarization and phase distributions, thereby enabling on-chip generation of single-photon skyrmions. We note that, in a recent study[31] reporting an important realization of single-photon skyrmions based on a quantum dot coupled to a Gaussian microcavity, mode selectivity was achieved through Zeeman splitting under low temperature and an external magnetic field. In contrast, our chip-integrated metasurface-QE platform is based on a different physical mechanism and operating regime, enabling room-temperature operation without external

magnetic fields and providing flexible access to a broader range of skyrmionic states through the dedicated metasurface design." – page 3

2) The authors have established a theoretical framework for generating tunable skyrmions using metasurfaces; however, further clarifications are required for the following points: a) The authors' previous work has demonstrated that concentric rings can modulate surface plasmon polaritons. It is recommended that the authors explain in the supplementary materials why L-shaped meta-atoms oriented at 45 can modulate surface plasmon polaritons with different polarization states, with the explanation tailored to the comprehension level of non-specialist readers. b) A mathematical expression (e.g., analogous to Eq. 1 for skyrmions) is lacking for skyrmionium. Although the authors provided the expression for the Laguerre-Gaussian (LG) superposition state in the supplementary materials, the decomposition results for skyrmionium were not presented. The authors should supplement this key theoretical content to ensure the completeness of their framework.

Our response:

We thank the reviewer for the comment and would like to enlarge as follows.

a) The L-shaped meta-atom is designed to couple the incident SPP field out into propagating away circularly polarized (CP) light, thereby introducing the spin angular momentum into outgoing free-propagating radiation. Taking advantage of the structural anisotropy (i.e., two notably elongated and orthogonal nanobricks separated by a quarter of the SPP wavelength distance, which results in a $\pi/2$ phase difference between the out-scattered SPPs), the dielectric meta-atom enables outcoupling of the in-plane SPP field component into highly directional CP emission, with the CP intensity crosstalk being < 1/125. In the revision, we have added a section - Supplementary Note S1 Meta-atom design for QE polarization control and Fig. 1 to introduce the detailed design principle.

b) In the revised manuscript, we have added the mathematical expression of generating the skyrmionium in Eq. (3) to ensure the completeness of our framework. Correspondingly, in the Fig.S4, we show the generated total intensity and the decomposition results, showing the separation of spatial mode for RCP and LCP components. The relevant descriptions have been added to elaborate this part to both the main and supporting manuscript parts:

"…Achieving this requires a tailored coupling between SAM and OAM, which can be constructed by the superposition of certain eigenstate of LG_0^1 with no radial mode ($\ell_\mathrm{R} = 1, p = 0$) and LG_1^{-1} with radial mode ($\ell_\mathrm{L} = -1, p = 1$), given by:

$$|\Psi(r)\rangle = \mathrm{LG}_0^1(r)|\mathrm{R}\rangle + \exp(i\theta)\,\mathrm{LG}_1^{-1}(r)|\mathrm{L}\rangle \tag{3}$$

The different radial distributions of mode fields reduce the spatial overlap between the RCP and LCP components.…" – Main text, page 6

"…Thus, the skyrmionium field can be constructed by superposing LG_0^1 and LG_1^{-1} modes with opposite circular polarizations:

$$|\Psi(r)\rangle = \mathrm{LG}_0^1(r)|\mathrm{R}\rangle + \exp(i\theta)\,\mathrm{LG}_1^{-1}(r)|\mathrm{L}\rangle \tag{S11}$$

Substituting Eqs. $S7$ and $S10$ into Eq. $S11$ yields the following expression for the skyrmionium field in the near field:

$$|\Psi(r,z=0)\rangle = \frac{\sqrt{2}}{w\sqrt{\pi}}\exp\left(-\frac{r^2}{w^2}\right)\cdot\left(\sqrt{2}\frac{r}{w}\right)\cdot\exp(i\varphi)\cdot\frac{1}{\sqrt{2}}\binom{1}{-i}+$$

$$+\exp(i\theta)\cdot\frac{1}{w\sqrt{\pi}}\exp\left(-\frac{r^2}{w^2}\right)\cdot\left(\sqrt{2}\frac{r}{w}\right)\cdot\left(2-\frac{2r^2}{w^2}\right)\exp(-i\varphi)\cdot\frac{1}{\sqrt{2}}\binom{1}{i}=$$

$$=\frac{1}{w\sqrt{\pi}}\exp\left(-\frac{r^2}{w^2}\right)\cdot\left(\sqrt{2}\frac{r}{w}\right)\left\{\exp(i\varphi)\cdot\binom{1}{-i}+\exp(i\theta)\cdot\left(2-\frac{2r^2}{w^2}\right)\cdot\exp(-i\varphi)\cdot\frac{1}{\sqrt{2}}\binom{1}{i}\right\} \quad (S12)$$

The top row of Supplementary Fig. 4 shows the calculated intensity distributions of the superposed field at $\theta = 0$. The corresponding decomposed components indicate the spatial separation of the RCP and LCP contributions. The resulting spatial polarization-vector distributions shown in the bottom row exhibit a nested polarization texture with double radial reversal, revealing a skyrmionium-like topology. As θ varies from $0, \pi/2$ to π, the skyrmionium field exhibit continuous rotation and internal twisting, corresponding to different helicity parameters ($\gamma = \pi, \pi/2, 0$)." – Supplementary page 13

3) The experimental data presented by the authors provide compelling evidence for the effective generation of single-photon skyrmions; nevertheless, certain details need to be supplemented to enhance the rigor and completeness of the results:

a) In Figure 2, the authors demonstrate the coupling of different orbital angular momentum (OAM) states with distinct polarization states. To better illustrate the deviation between the experimental and theoretical values of N, the authors should provide the OAM spectral distribution in the measured OAM space. Additionally, phase is a crucial parameter governing skyrmion types; thus, the authors should measure the relative phase and its associated uncertainty. This data can be obtained via projection measurements of the polarization and OAM degrees of freedom using the experimental setup described in the supplementary materials.
b) Similarly, the relative error for g(0) was not provided. The authors should supplement this error analysis to ensure the reliability and reproducibility of the experimental results.

Our response:

We thank the reviewer for the helpful comments and would like to substantiate as follows.

a) The OAM states were experimentally verified by projecting the emitted photons onto a set of OAM basis states using SLM phase masks under both RCP and LCP bases. As the reviewer suggested, we analysed the resulting OAM distribution, which has been added as Supplementary Fig. 9. The measured mode weights confirm that the target OAM modes, $\ell_R = 0$ and $\ell_L = -2$, remain dominant, with purities of 60% and 59%, respectively. The moderate reduction compared with the simulated values may arise from the wavelength-dependent response of the SLM under the broadband emission of NV centres, together with environmental photon noise during the measurement and minor fabrication imperfections of the device.
Furthermore, following the reviewer's suggestion, we analysed the spatially resolved relative phase distribution of the generated field. The phase was retrieved from the measured Stokes parameters according to $\phi = \mathrm{atan2}(S_2, S_1) + \pi$, with S_1 and S_2 reconstructed from the polarization-resolved intensity measurements (following the method used in Ref. [41]). In the revised manuscript, we have

added the Supplementary Fig. 10 - Simulated and experimental far-field intensity and spatially resolved relative phase distributions, which reveals the expected 4π azimuthal phase winding between LCP and RCP modes, providing further support for the targeted skyrmion topology.

More discussions have been added to the main text:
"To further analyse the distinct OAM modes, the RCP and LCP components were separately projected onto a spatial light modulator (SLM) encoded with $LG_0^{\ell_{SLM}}$ phase holograms with $\ell_{SLM} = -1, 0, 1, 2, 3$ (Fig. 2f). Without any additional phase modulation ($\ell_{SLM} = 0$), the emission appears as a central spot in the RCP channel and a doughnut-shaped pattern in the LCP channel, both being consistent with the targeted spatial mode profiles shown in Fig. 2c. When the OAM of the emitted photon is compensated by the conjugate OAM phase encoded on the SLM, the diffracted beam is converted into a Gaussian-like spot. Accordingly, bright Gaussian spots are observed at $\ell_{SLM} = 0$ for the RCP component and at $\ell_{SLM} = 2$ for the LCP component, confirming the topological charges of $\ell_R = 0$ and $\ell_L = -2$, respectively. Deviations in the measured OAM mode distribution from the simulated one (Supplementary Fig. 9) may arise from the wavelength-dependent response of the SLM to the broadband emission of NV centres, together with environmental photon noise during the measurement and minor fabrication imperfections of the component. Furthermore, we have also analysed the corresponding spatially resolved relative phase distribution between the two spin-orbit components, retrieved from the Stokes parameters[41], and observed a 4π azimuthal phase winding (Supplementary Fig. 10), providing further support for the targeted skyrmion topology." – Page 5

b) In the revision, we have added a quantitative uncertainty analysis for the measured $g^2(0)$. The corresponding uncertainty has now been added in the main text and Fig. 4b.
"The second-order correlation measurement a Hanbury Brown-Twiss configuration yields $g^2(0) = 0.19 \pm 0.04$, confirming the single-photon emission at room temperature (Fig. 4b)." – page 6
"The uncertainty was estimated by Poisson bootstrap resampling of the coincidence histogram." – Methods, page 8

Reviewer #2

In this manuscript Liu et. al propose and implement a metasurface coupled with quantum emitters (QEs) to create single-photon emission with skyrmionic polarization states. Optical skyrmions are, in general, a rich playground for topological photonics. Creation of various skyrmions has seen enormous effort in a variety of systems, including photonics, acoustics and magnetic systems. For free-space optical skyrmions, the first key step for such a system is to engineer distinct orbital angular momentum (OAM) modes with orthogonal polarizations. The authors employ a metasurface composed of two distinct types of meta-atoms, designed to generate individual Laguerre-Gaussian (LG) modes with right circular polarization (RCP) and left circular polarization (LCP), respectively. By superposing these two modes and coupling them to QEs, the emission states of the single photons can be controlled to obtain skyrmionic polarization distributions.
While I think the sample fabrication and QE implementation in this work are technically sophisticated, the authors don't convincingly demonstrate conceptual and theoretical novelty. For these reasons I cannot recommend this manuscript for publication as I found it incremental and does not meet the high standard of Nature Communications. I describe my reservations in more detail below.
<u>Our response:</u>
We thank the reviewer for raising this concern. We agree that the conceptual novelty was not stated clearly enough in the originally submitted version. Below, we take this chance to clarify the key advances of the present work over previous studies.

First, a note on the generation of arbitrary OAM modes and polarizations using metasurfaces: There are tremendous design strategies for optical metasurfaces that can generate various modes of OAMs and polarizations, including the LG modes with RCP/LCP polarizations. In this work, the key to generating a single-photon emission with a skyrmionic polarization distribution is the precise excitation of specific LG modes, the requirements for which are well established in Ref. [29] cited by the authors.
<u>Our response:</u>
We agree with the reviewer that metasurfaces have been widely used to generate optical fields with tailored OAM and polarization, and that the modal conditions for forming skyrmionic textures are established in free-space optics. However, most current skyrmion generation methods rely on bulk optics, such as interferometric superpositions and spatial light modulators (as the reviewer mentioned Ref. 29, now Ref. 30), to synthesize orthogonally polarized modes with tailored OAM, which inherently restricts system stability and scalability. While some progress has been made toward integration, these approaches involve external free-space illumination and are therefore not directly applicable to the task of shaping the emission from nanoscale single-photon sources. So far, the on-chip generation of single-photon skyrmions remains elusive and at a very early stage. For our work, rather than shaping externally incident <u>classical light</u>, we directly, on-chip, generate skyrmionic <u>single-photon emission</u> from a nanoscale quantum emitter. This is a fundamentally different problem, because the emitter is highly localized and thus generates strongly divergent waves, if in free space, making direct at-source control of the emitted topological state highly challenging.
To the best of our knowledge, the only previous on-chip experimental realization of single-photon skyrmions from a QE is Ref. 31, which relies on a quantum dot coupled to a Gaussian microcavity

and operated at low temperature and under an external magnetic field. By contrast, our work realizes single-photon skyrmions by chip-integrated metasurface-QE platform at room temperature and without magnetic fields. Our conceptual advance is to use QE-excited SPPs as an intermediate channel between the emitter and the metasurface, thereby enabling the required skyrmion-forming mode engineering in an emitter-integrated architecture. In this sense, the present work progresses from free-space bulk skyrmion optics toward quantum skyrmion emission from nanoscale solid-state emitters on a chip.

We have added the following clarification in the revised manuscript:

"…Despite these advances in classical optics[25–27], the extension of such topological field configurations to the quantum regime remains at an early stage. ." – page 1

…However, realizing quantum skyrmions in integrated photonic platforms without relying on complex optical components or stringent operating conditions remains highly challenging, particularly for nanoscale solid-state quantum emitters (QEs)[31,32]…." – page 2

"…By engineering arrays of subwavelength meta-atoms, we realize diverse skyrmionic single-photon states from QEs without relying on bulky optical systems, external magnetic fields, or cryogenic operation, thereby establishing a versatile on-chip platform for generating and exploring topologically structured quantum light for advanced quantum technologies." – page 2

Second, a note on the implementation of QEs in the color center of a metasurface: I found very similar works reported by some of the authors of the current manuscript demonstrating respectively:

A) (as in Ref. [32], Kan, Y. et al. Adv. Mater. 32, 1907832 (2020)) dipolar QEs placed in the color center and coupled to surface plasmon polariton (SPP) modes of a metasurface composed of circular nanoridges, which upon interaction with an optical metasurface radiate into free space in the form of a collimated single photon stream carrying the designed chirality, i.e., RCP or LCP;

B) (as in Ref. [26], Kan, Y. et al. Sci. Adv. 10, eadq6298 (2024)) QEs placed in the color center of Moire metasurfaces enable the generation of structured single photon emission vortex beams with vectorial spin-orbital states. Similarly, the control of the emission state is through coupling the QEs with RCP and LCP, each carrying two different OAMs.

<u>Our response:</u>

We appreciate the reviewer for raising the comparison with our previous works in Refs. 26 and 32 (now Refs. 27 and 34). These earlier works demonstrated the possibility of the emitter-integrated metasurfaces for shaping of quantum emission. However, the present work goes beyond those demonstrations in both objective and design requirements. In the previous work, the primary goal was to generate selected polarization and OAM of emitted single photons with the assistance of nanoridges. In contrast, generating skyrmionic polarization textures requires a more restrictive and spatially organized modal condition: the emitted field must be constructed from spatially distributed orthogonal basis states with well-defined amplitude, phase, and polarization relations, such that the local polarization continuously evolves and covers the Poincaré sphere in a topologically nontrivial manner. This is therefore not a straightforward extension of producing a photon with a chosen chirality or OAM, but requires a different design strategy aimed at engineering the full polarization topology of the emitted field.

For this reason, in the present work we introduced anisotropic meta-atoms with opposite chirality and designed their spatial trajectories specifically to generate orthogonally polarized LG modes that satisfy the specific skyrmion-forming conditions, such as those expressed by Eq. (1) and Eq. (3), the latter is newly added following the recommendation of Reviewer #1. This allowed us not only to realize room-temperature on-chip single-photon skyrmions, but also to access multiple topological states, including high-order anti-skyrmions and skyrmionium. We therefore view the present manuscript as a significant conceptual advance from structured single-photon emission toward topological single-photon emission.

To clarify this distinction, we have added the following discussion in the revised manuscript:

"The skyrmionic beam features a spatially varying polarization texture that wraps the Poincaré sphere. Here, the composite metasurface is composed of two surface domains containing meta-atoms of opposite chirality, with the chirality of meta-atoms controlling the spin angular momentum (SAM) components of emitted photons and their spatial arrangement controlling the corresponding orbital angular momentum (OAM) and spatial distribution. In this way, the metasurface constructs two spatially distributed orthogonal basis modes, represented as the poles of a higher-order Poincaré sphere (HOPS), whose superposition gives rise to the skyrmionic topology (Fig. 1b). This goes beyond the generation of single-photon emission with selected polarizations or OAMs[30,34], requiring instead a conceptually different design strategy that establishes well-defined spatial amplitude, phase, polarization, and radial-mode relations between the two basis modes that enable the local Stokes vector to continuously evolve in the skyrmionic beam cross section, uniformly covering the Poincaré sphere.…" – page 2-3

Therefore, the combination of these two concepts - metasurface-based LG mode generation and QE-excited SPPs appears to be an incremental development, which may not meet the high bar of scientific significance set by Nature Communications, although the presentation is technically sound and the correctness of the results is unquestionable.

Our response:

With the above clarifications, we hope it is now clear that the present work is not an incremental combination of existing elements, but the establishment of an emitter-integrated metasurface design strategy for topological single-photon emission. Conceptually, in this strategy, meta-atom chirality controls the polarization components, spatial arrangement controls the OAM and field distribution, providing the spatial freedom required to directly engineer real-space skyrmionic polarization textures, including anti-skyrmions, high-order anti-skyrmions, and skyrmionium. Experimentally, this is demonstrated on chip, at room temperature, without external magnetic fields, and across multiple topological states and different color-center-in-diamond emitters. We have revised the manuscript accordingly to make these conceptual and experimental advances more explicit, and hope that the significance of our work is now sufficiently articulated.

Reviewer #3

I co-reviewed this manuscript with one of the reviewers who provided the listed reports. This is part of the Nature Communications initiative to facilitate training in peer review and to provide appropriate recognition for Early Career Researchers who co-review manuscripts.

Our response:

We thank the reviewer for careful evaluation of our manuscript and the constructive comments.

Reviewer #4

In this manuscript, the authors report room temperature, on-chip generation of single-photon skyrmions using a metasurface-integrated quantum emitter platform. By engineering arrays of subwavelength meta-atoms, the authors shape the emission from different colour centres in nanodiamonds into diverse skyrmionic single-photon states via spin-orbit interaction.

Overall, the work is interesting and timely. The fabrication, optical characterization, and experimental control appear technically sound. However, there are technical issues preventing its publication in the current form.

Our response:

We appreciate the overall positive evaluation noting its novelty and significance.

1. The manuscript emphasizes "on-chip generation, however, the Introduction would benefit from a more explicit discussion of recent integrated optical skyrmion source demonstrations (e.g., micro-ring (Optica, 11, 1588(2024)), q-plate (Phys. Rev. Lett., 134, 083802 (2025)), bullseye cavity (Nanophotonics, 14(24), 4411 (2025), metasurface (Nanophotonics, 14(23), 4069 (2025))).

Our response:

We thank the reviewer for this helpful suggestion. We have revised the Introduction accordingly and added these recent relevant demonstrations.

"The free-space skyrmionic beams exhibiting unique features such as space-time nonseparability and topological resilience in disordered media or free-space propagation[18–20]. More recently, integrated optical platforms have also been explored for generating skyrmionic light, including micro-rings,[21] q-plates,[22] and metasurfaces[23,24]. …" – page 1

2. In lines 39-40, the authors state that "current implementations typically rely on complex optical setups, or require strict operational conditions, which hinder scalability and tunability". However, the cited references (ref. 20, 27-29) at that location appear not fully aligned with the single-photon skyrmion context.

Our response:

We thank the reviewer for this important comment. We agree that the previous wording could be misleading, and we have therefore revised the sentence to make this distinction explicit.

"Despite these advances in classical optics[25–27], the extension of such topological field configurations to the quantum regime remains at an early stage." – page 1

"However, realizing quantum skyrmions in integrated photonic platforms without relying on complex optical components or stringent operating conditions remains highly challenging, particularly for nanoscale solid-state quantum emitters (QEs)[31,32]…." – page 2

3. The authors state that the metasurface was "precisely integrated" with a nanodiamond containing NV centres (lines 109-112). Please provide a quantitative estimate of the nanodiamond positioning accuracy, and discuss how misalignment affects the quality of the emitted single-photon skyrmions.

Our response:

In the revised manuscript, we have evaluated the relative positioning accuracy with the help of the SEM image. By overlaying the SEM image of the fabricated structure with the corresponding design

layout (added in Supplementary Fig. 8a), we estimate the positioning shift to be approximately 20 nm, taking into account the practical size of the nanodiamond. To further address the reviewer's concern, we have performed simulations to evaluate the effect of QE misalignment on the performance. We consider QE displacements along the x direction as well as in both x and y directions (Supplementary Fig. 8b). The results show that the skyrmion number decreases monotonically with increasing displacement, while remaining about -1.9 for lateral offsets with 30 nm.

In the revision, we provide a more detailed explanation of fabrication-induced deviations, also noting the influence of the position shift on the performance:

"The precision in relative lateral positioning of the ND with respect to the metasurface is estimated to be ~20 nm." – page 4

"…The deviation from the ideal skyrmion number (−2) mainly originates from the non-uniform far-field intensity distribution in the experiment, which affects the robustness of the retrieved Stokes parameters in weak-intensity regions. This non-uniformity may arise from the ND position shift, as well as fabrication imperfections. Based on the analysis of the influence of ND positioning accuracy (Supplementary Fig. 8), the skyrmion number decreases monotonically with increasing displacement, while remaining about -1.9 for lateral offsets with 30 nm. Nevertheless, the main skyrmionic textures are preserved within this moderate displacement range, indicating robust tolerance to positioning imperfections." – page 5

4. The skyrmion number is defined in Eq.(2) as a 2D integral over x,y, but the integration domain is not specified in Eq.(2). In addition, the authors should explain how the skyrmion regions in Fig.3(d,f) and Fig.4(c,d) are determined.

Our response:

We appreciate the concern expressed. The integration domain in Eq. (2) is defined as the circular region where the total far-field intensity exceeds $1/e^2$ of its maximum value. This region is taken as the effective area of the emitted field for evaluating the skyrmion number, similarly to what is done when evaluating the Gaussian beam diameter. The region also helps minimize the influence of noise from low-intensity areas.

The integration regions are consistent with the circled far-field intensity areas shown in the inset of Fig. 4(c,d). The same criterion is applied to Fig. 3(d,f), where the corresponding integration areas are also encircled. Momentum-space labels are added to facilitate comparison with the far-field intensity distribution.

In the revised manuscript, the corresponding discussions have been added to make the definition of integration domain clearer to the reader:

"Here, the integration domain for evaluating the skyrmion number is defined as the circular region where the total far-field intensity exceeds $1/e^2$ of its maximum value, a criterion that is widely used for evaluating the Gaussian beam diameter. The obtained polarization field indicates the higher order anti-type skyrmion distributions with the simulated skyrmion number of -1.98." – page 4

"…The measured skyrmion number is −1.86, evaluated over an integration region based on the far-field intensity distribution shown in the inset, within a polar angle of 9°…" – page 4

"Fig.4 **e,** Simulated and **f**, Measured polarized distribution of the emitted single photons, exhibiting a complete transition from RCP to LCP back to RCP. The integration region used to calculate the

skyrmion number follows the corresponding far-field intensity profiles, with NA = 0.14 (e) and NA = 0.15 (f)." – page 16

5. The presentation of the results in Fig. 2 is somewhat confusing. (i) Please clarify whether Fig.2(c) shows simulated or experimental data. (ii) The manuscript reports a measured skyrmion number of -1.86, but it is not clearly stated which measurement this value is extracted from (presumably Fig.2(e)). In addition, the ideal value is -2. Please provide a quantitative analysis of the discrepancy.

Our response:

We thank for the important comment. Fig. 2(c) shows the design principle with the evolution of skyrmionic emission with different helicities, where the insets are obtained from the simulations. The measured skyrmion number is obtained from Fig.2(e). The discrepancy may mainly arise from the discontinuous intensity distribution in the measured far field. Therefore, some weak-intensity areas are included in the evaluation, where the extracted Stokes parameters are more susceptible to fluctuations and noise. This affects the experimentally reconstructed polarization field and therefore leads to a deviation of the extracted skyrmion number from the ideal value. The observed intensity inhomogeneity is likely related to practical imperfections of the nanostructure and slight displacement of the quantum emitter, both of which can distort the ideal emission pattern.

The following discussion has been added to the revised manuscript:

"Both simulated (Fig. 2d) and measured (Fig. 2e) polarization states show a progressive change of the vector orientation from the 'up' state at the centre to the 'down' state at the edge, covering all intermediate polarization states from RCP to LCP. The measured skyrmion number is −1.86, evaluated over an integration region based on the far-field intensity distribution shown in the inset, within a polar angle of 9°. The deviation from the ideal skyrmion number (-2) mainly originates from the non-uniform far-field intensity distribution measured in the experiment, which affects the robustness of the retrieved Stokes parameters in weak-intensity regions. This non-uniformity may arise from the ND position shift, as well as fabrication imperfections. Based on the analysis of the influence of ND positioning accuracy (Supplementary Fig. 8), the skyrmion number decreases monotonically with increasing displacement, while remaining about -1.9 for lateral offsets with 30 nm. Nevertheless, the main skyrmionic textures are preserved within this moderate displacement range, indicating robust tolerance to positioning imperfections." – page 4-5

6. In Fig.3(c,e), the authors aim to generate LG modes where the RCP component is described as having no radial mode. However, an additional ring-like feature appears in the measured intensity distribution. This extra ring could potentially introduce an extra polarization flip along the radial direction. Please explain this feature.

Our response:

We thank the reviewer for this insightful comment. The weak outer ring observed in the measured RCP intensity distribution is not part of the ideally designed LG mode with zero radial order. A possible origin of this feature might be related to the residual SPPs in the gap between neighbouring sections of the metasurface, which are not fully outcoupled in the designed region and may therefore undergo additional scattering. To further clarify this effect, we investigated metasurfaces composed only of RCP units and analysed the corresponding emission patterns for different ring numbers. As

shown in the added Supplementary Fig. 12, the extra far-field intensity ring is gradually suppressed as the metasurface ring number increases. This observation shows that additional grating rings enhance the outcoupling of the residual SPP field and thereby suppress the unwanted outer-ring intensity feature. In the skyrmionium design, this contribution could in principle be further reduced by decreasing the gap between the second and third rings, which is expected to improve the outcoupling of the remaining SPP component. This trend is consistent with the results shown in Supplementary Fig. 11, in which smaller gaps show clearer patterns. We have added more discussion in the revised manuscript to clarify the possible origin of this feature.

"A weak outer ring seen in the RCP intensity distribution may originate from residual SPPs in the gap between neighbouring rings of opposite handedness, and can progressively be suppressed as the gap is reduced (for example, by decreasing r_{L2}; Supplementary Fig. 11) or as the number of RCP rings (Supplementary Fig. 12) is increased, owing to more efficient SPP outcoupling." – page 6

7. In Fig.4(d), the measured S3 distribution deviates from the expected circular symmetry and exhibits a clear two-lobed pattern. This could distort the skyrmion texture and thus influence the extracted skyrmion number. Please comment on the origin of this symmetry breaking and quantify its impact on the reconstructed skyrmion texture and the calculated skyrmion number.

<u>Our response:</u>

We thank for the comment. In practice, achieving a perfectly symmetric circular polarization pattern is always challenging, especially in single-photon measurements, where the relatively weak signal is more susceptible to noise and other experimental imperfections. In this work, the distortion of the skyrmionium texture may originate from several reasons, including non-ideal radiative dipole orientation in the quantum emitter, imperfections in metasurface geometry, possible QE position shift, and additional distortions introduced during the measurement process after a weak signal passes through multiple optical components. These experimental imperfections can jointly affect the ideal circular symmetry and lead to the observed deformation of the polarization texture.

Nevertheless, although distortion and intensity inhomogeneity observed, Fig. 4f still clearly demonstrates the nontrivial skyrmion texture, demonstrating the robustness of our design to experimental imperfections. In the revised manuscript, we have added further discussion of these results as follows:

"…The minor deviations and the slight rotation of the Stokes pattern may arise from fabrication-induced asymmetries of constituent meta-atoms and an imperfect (i.e., deviating from normal to the surface plane) radiative dipole orientation of the colour centre, which lead to unequal RCP/LCP amplitudes and an intensity discontinuity at the lobe boundary (inset of Fig. 4f). In addition, inherently weak single-photon signals may further be attenuated after passing through multiple optical components during the measurement procedure, making the above effects more pronounced. Nevertheless, the nontrivial skyrmion texture remains clearly visible, indicating that the proposed design can reliably preserve the key skyrmionic features.…" – page 6-7

8. The reported g2(0) 0.19 lacks uncertainty estimates.

<u>Our response:</u>

Thanks for the comment. We have added the uncertainty estimate for $g^{(2)}(0)$in the revised manuscript. The uncertainty was obtained by Poisson bootstrap resampling of the coincidence histogram followed by repeated fitting using the same model, yielding $g^{(2)}(0) = 0.19 \pm 0.04$.

9. In several figures, the colorbars indicate numerical ranges, but the underlying physical quantity represented by the color scale is not specified. In addition, some of these panels lack clear coordinate axes, making it difficult to interpret the spatial scale. This issue appears in Fig.2(c-f), Fig.3(c-f), and Fig.4(c-f).

Our response:

Thanks for the comment. We have added the physical quantity and coordinate axes in the revised figures.

Reviewer #5

I co-reviewed this manuscript with one of the reviewers who provided the listed reports. This is part of the Nature Communications initiative to facilitate training in peer review and to provide appropriate recognition for Early Career Researchers who co-review manuscripts.

Our response:

We thank the reviewer for careful evaluation of our manuscript and the constructive comments.

Concluding, the authors would like to again thank all reviewers for professional and valuable comments that were very helpful in improving the clarity and completeness of the manuscript. With the above detailed clarifications and additional discussions introduced, we hope that our revised manuscript will be found suitable for the publication in *Nature Communications*.
